# Neural Correlates of Moral Judgment in Dilemma Versus Non‐Dilemma Contexts

**DOI:** 10.1002/brb3.71864

**Published:** 2026-10-21

**Authors:** Junjun Fu, Di Song, Serenella Tolomeo, Rongjun Yu

**Affiliations:** ^1^ School of Psychology Key Laboratory of Behavioral and Mental Health of Gansu Province Northwest Normal University Lanzhou China; ^2^ Department of Education and Psychology Academy of Wellness and Human Development Hong Kong Baptist University Hong Kong China; ^3^ Institute of Advanced Intelligence and Computing (IAIC) Agency For Science, Technology and Research (A*STAR) Singapore Singapore; ^4^ Life Science Imaging Center Hong Kong Baptist University Hong Kong China

**Keywords:** ALE, fMRI, moral judgment, moral dilemma

## Abstract

**Purpose:**

Moral dilemmas have been extensively used to probe the hierarchy of moral preferences. However, human moral judgment frequently operates not only through competing principles but also in straightforward transgression contexts that do not constitute a dilemma. Consequently, it is unclear whether the moral circuitry is differentially engaged in dilemma‐based versus non‐dilemma‐based moral processing.

**Method:**

We synthesized neuroimaging studies of dilemma‐based moral tasks and non‐dilemma moral tasks. Activation likelihood estimation revealed shared regions including the medial prefrontal cortex and temporoparietal junction (TPJ).

**Findings:**

Non‐dilemma tasks showed greater engagement of the left ventromedial prefrontal cortex, left orbitofrontal cortex, right temporal pole, and right precuneus, regions that have been implicated in affective valuation and rapid social appraisal. In contrast, moral dilemma tasks showed greater engagement of the left dorsomedial prefrontal cortex and bilateral TPJ, regions commonly associated with conflict monitoring and mental‐state inference. Meta‐analytical connectivity modeling indicated salience, default mode, and limbic network engagement during non‐dilemma tasks, with additional central executive network recruitment during dilemma tasks. Activation network mapping revealed that both task types engage a common mesocorticolimbic connectivity network.

**Conclusion:**

These findings indicate that non‐dilemma and dilemma‐based moral tasks share a common neural core while showing differential patterns of regional involvement that may reflect their differing cognitive and affective demands.

## Abstract 2

This article revealed that non‐dilemma and dilemma‐based moral tasks share a common neural core, yet each engages distinct brain regions in different ways.

## 1 Introduction

The long‐standing debate over the relative roles of reason and affect in morality has attracted sustained attention from different research fields, such as philosophy (Kant 1785/1993; Hume 1777/1969), psychology (Kohlberg 1963; Haidt 2001), and neuroscience (Greene et al. 2001). Rationalist accounts emphasize the deliberate application of moral principles (Kant 1785/1993), whereas social intuitionist models highlight rapid, emotion‐driven moral responses (Haidt 2001). A theoretical perspective proposes that the contribution of reason versus affect depends on the moral situation (Monin et al. 2007). Moral dilemma scenarios that pit competing principles, such as obeying the law versus saving a loved one in Kohlberg's classic task, encourage careful consideration of competing principles and consequences. These vignettes typically prompt participants to provide first‐person guidance to the actor through questions such as “Do you … to …?” (e.g., Cecchetto et al. 2019), “Is it appropriate for you to …?” (e.g., Greene et al. 2004), or “Is it OK for … to do this?” (e.g., Reniers et al. 2012). In contrast, non‐dilemma moral vignettes usually present clear transgressions, such as killing someone for money (e.g., Greene et al. 2001), and ask participants to judge the rightness or wrongness of the transgressors’ conduct, such as “Is it right?” (e.g., Avram et al. 2013; Prehn et al. 2008), allowing responses guided more by immediate moral emotions such as disgust or contempt (Moll et al. 2002a) rather than by complex reasoning. Thus, dilemma‐based moral judgment tasks require participants to resolve conflicts between competing moral principles (e.g., utilitarian versus deontological choices) or conceptually equivalent paradigms in the dilemma moral judgment context. In contrast, non‐dilemma moral judgment contexts do not involve competing moral principles but instead involve evaluating the moral acceptability of actions, statements, or behaviors.

Despite the widespread use of both dilemma and non‐dilemma tasks, the neuropsychological mechanisms that differentiate their processing remain poorly specified. In particular, the existing literature still provides limited empirical evidence as to whether moral dilemma tasks demand relatively more deliberative processing, whereas non‐dilemma tasks rely more on intuitive affect. Addressing this question can help reconcile theoretical debates concerning cognition and emotion in morality and provide a more nuanced understanding of how moral decisions are made across different contexts.

Existing neuroimaging studies have separately investigated the neural correlates of moral decision‐making using non‐dilemmatic (Heekeren et al. 2005; Heekeren et al. 2003a; Luo et al. 2006; Moll et al. 2002a, 2002b) and dilemma‐based moral tasks (Borg et al., 2006; Greene et al., 2001, 2004). These studies consistently report the involvement of a functional network including the ventromedial prefrontal cortex (vmPFC), temporoparietal junction (TPJ), orbitofrontal cortex (OFC), temporal pole (TP), amygdala, posterior cingulate cortex (PCC), and posterior superior temporal sulcus (pSTS)—regions implicated in both affective and cognitive aspects of moral processing (Casebeer 2003; Casebeer and Churchland 2003; Goodenough and Prehn 2004; Lieberman 2007; Moll et al. 2003; Moll et al. 2005). Key nodes of the default mode network (DMN), such as the medial prefrontal cortex (mPFC), precuneus, and lateral parietal cortex (Buckholtz and Marois 2012; Fox et al. 2005; Raichle et al. 2001), also feature prominently, reflecting contributions from autobiographical memory retrieval and mentalizing (Andrews‐Hanna et al. 2014; Buckner et al. 2008; Spreng and Grady 2010).

One meta‐analysis investigated the convergent and divergent brain processes underlying moral reasoning and moral emotion (Sevinc and Spreng 2014). This study classified tasks requiring explicit moral judgment (e.g., “Please rate the permissibility of the moral action”) as moral reasoning, and tasks involving passive exposure to moral stimuli without a required moral‐related reaction as moral emotion. Active moral judgments elicited greater activity in the TPJ, angular gyrus, and TP compared with passive viewing. Although this study did not directly contrast dilemma and non‐dilemma moral tasks, the results show that the relative extent to which affect and cognition contribute to morality depends on task demands.

Another meta‐analysis compared brain activity during moral response decisions, which involve making moral decisions, and moral evaluations, which involve judging others’ moral actions (Garrigan et al. 2017). Although both task categories included moral dilemma and non‐dilemma scenarios, response decision tasks typically require first‐person choices characteristic of moral dilemmas (e.g., “Would you … in order to …?”), whereas moral evaluation tasks require participants to judge others’ moral behaviors from a third‐party perspective, as is common in non‐dilemma tasks (e.g., “Is it wrong?”). Contrast analysis showed that the lateral middle temporal gyrus (MTG) and the right precuneus were additionally activated during moral response decisions compared with moral evaluation, demonstrating that making moral decisions from a first‐person perspective recruits different brain regions from those involved in judging others’ moral conduct from a third‐person viewpoint.

The current study directly compares the neural correlates of moral decision‐making across dilemmatic and non‐dilemmatic vignettes at two complementary levels: regional and network. At the regional level, we conducted activation likelihood estimation (ALE) meta‐analyses for each task type, alongside conjunction and contrast analyses to identify shared and distinct brain regions. Given the role of emotion in guiding adaptive behavior in complex social contexts (Anderson et al. 1999; Damasio 1996), we further categorized contrasts by scenario valence (positive, negative, mixed) and performed sub‐analyses. At the network level, we used meta‐analytic connectivity modeling (MACM) to characterize co‐activation patterns and activation network mapping (ANM) to capture reproducible functional networks associated with each task (Peng et al. 2022). Finally, functional decoding analyses were conducted to examine the functional significance of identified regions.

## 2 Materials and Methods

### 2.1 Meta‐analysis

#### 2.1.1 Literature Search and Article Selection

A systematic database search was first conducted in September 2024 on PubMed (https://www.ncbi.nlm.nih.gov/pubmed), Web of Science (www.webofknowledge.com), and ScienceDirect (https://www.sciencedirect.com/), following the Preferred Reporting Items for Systematic Reviews and Meta‐Analyses (PRISMA) guidelines (Moher et al. 2009). The search was updated in January 2026. The following terms were used: [“Moral” OR “Moral judgment” OR “Moral evaluation” OR “Moral decision‐making” OR “Moral reasoning” OR “Moral dilemma” OR “Ethical decision‐making”] AND [“functional magnetic resonance imaging” OR “fMRI” OR “positron emission tomography” OR “PET”]. In addition, we searched additional sources, including the reference lists of previously published reviews, meta‐analyses, and pre‐selected articles. Finally, we used Google Scholar (https://scholar.google.com.sg/) to search for additional related articles by entering the same key terms mentioned above.

The search yielded 712 potentially relevant articles, which were eligible if they satisfied the following criteria: (1) reported contrasts (e.g., moral decision vs. non‐moral decision/control, moral dilemma vs. non‐moral dilemma/control) using whole‐brain general linear model analyses; (2) reported one of the previously mentioned contrasts in healthy participants without any psychiatric or neurological disorders; (3) used fMRI or PET as the imaging modality; (4) presented activations as Talairach or Montreal Neurological Institute (MNI) coordinates in a standardized stereotaxic space (Holmes et al. 1996). Note that we transformed the focus coordinates reported in Talairach space into MNI space using the Lancaster transformation (Lancaster et al. 2007). Following published guidelines (Müller et al. 2018), to minimize the risk of selection bias and ensure the quality of inclusion, the stepwise screening process was conducted independently by two investigators (J.F. and D.S.), who worked with regular meetings. Any disagreements between them were adjudicated by a third reviewer (S.T.). The final set of included studies was determined through consensus, yielding 100% agreement following discussion.

After screening articles using our eligibility criteria, we included 50 articles: 28 on non‐dilemma moral tasks [32 contrasts (13 positive content valences; 12 negative content valences; 7 mixed content valences); 279 foci; 755 subjects] and 22 on dilemma moral tasks [30 contrasts (30 negative content valences); 250 foci; 713 participants]. Figure 1 shows PRISMA flowcharts (Page et al. 2021) with the specific steps taken to identify eligible fMRI studies for non‐dilemma and dilemma moral tasks; no PET studies met the inclusion criteria. Tables 1 and 2 summarize the eligible contrasts for each meta‐analysis. Supplementary materials detail dilemma status, stimulus source, scenario type, perspective (first‐/third‐person), response format, stimulus valence, control condition, and all contrasts and peak coordinates (see Supplementary Materials Tables S1–S4; https://doi.org/10.17605/OSF.IO/X6AEW).

**FIGURE 1:**
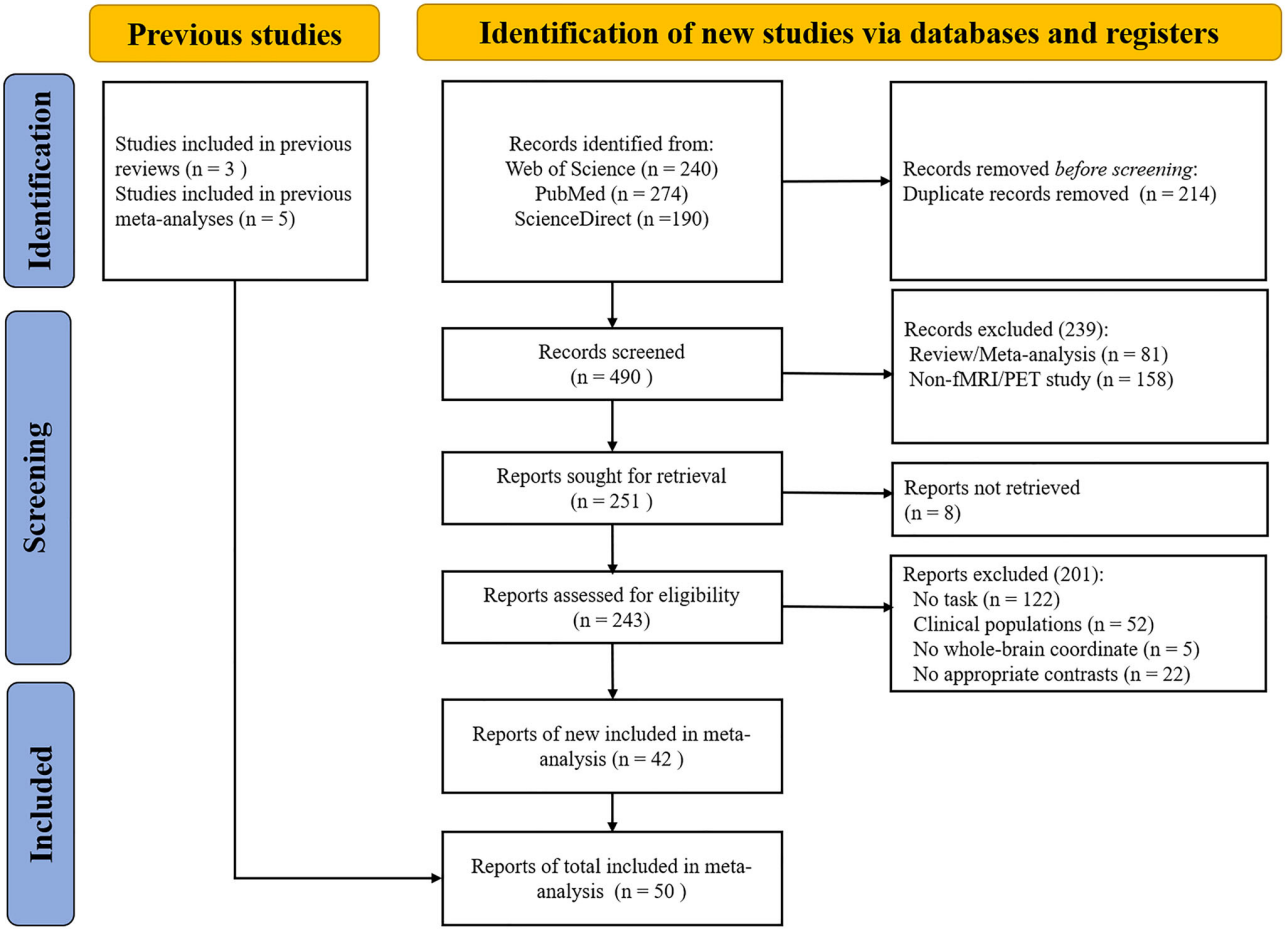
PRISMA flowchart. *n*, number of articles; fMRI, functional magnetic resonance imaging; PET, positron emission tomography.

**TABLE 1:** Summary of studies included for the meta‐analysis of the non‐dilemma moral task (*n* = 32).

Article	*N* (female)	Mean age (*SD*)	Age range	Stimuli	Contrast	Foci	SPACE	SOFT	Tesla
Avram et al. (2013)	16 (9)	28.25 (NA)	NA	Statements	Moral judgment > esthetic judgment	8	MNI	SPM8	3.0
Avram et al. (2014)	16 (9)	28.25 (NA)	NA	Statements	1pp > non‐moral	5	MNI	SPM8	3.0
					3pp > non‐moral	5			
Bahnemann et al. (2010)	25 (3)	26 (4.43)	NA	Scenarios	Moral judgment > physical norm judgment	11	MNI	FEAT	1.5
Chen et al. (2020)	40 (21)	23.5 (2.2)	20‐36	Scenarios	Helping > harming	3	MNI	SPM12	3.0
Cheng et al. (2020)	25 (25)	21 (1.7)	NA	Scenarios	Moral beauty > bad Behavior ratings	9	MNI	SPM12	3.0
					Moral goodness ratings > bad behavior ratings	2			
FeldmanHall et al. (2012)	14 (8)	25.9 (4.6)	NA	Scenarios	Real PvG > non‐moral PvG	4	MNI	NA	3.0
Harada et al. (2009)	18 (10)	25.3(4.2)	NA	Scenarios	Morality judgment > control condition	9	MNI	SPM2	3.0
Harenski et al. (2008)	28 (14)	25.2 (NA)	NA	Scenarios	Moral > non‐moral	15	MNI	SPM2	3.0
Harenski et al. (2012)	36 (NA)	27.3 (8.37)	19‐53	Scenarios	Moral > non‐moral	11	MNI	SPM5	1.5
Heekeren et al. (2003)	8 (1)	27.8 (4.5)	NA	Statements	Moral judgment > semantic judgment	9	Talairach	BrainVoyager 2000	1.5
Heekeren et al. (2005)	12 (2)	25.75 (1.54)	NA	Statements	Moral decision > semantic decision	8	MNI	FEAT	1.5
Li et al. (2016)	24 (12)	20.42 (1.06)	NA	Statements	Immoral intention > neutral	4	MNI	SPM8	3.0
Liu et al. (2019)	24 (13)	22.7 (2.8)	NA	Statements	IM_Hp > IM_Lp	15	MNI	SPM12	3.0
Moll et al. (2001)	10 (4)	NA (NA)	24‐43	Statements	Moral > factual	10	Talairach	BrainVoyager 1999 version 3.9	1.5
Moll et al. (2002a)	7 (4)	30.3 (4.7)	NA	Statements	Moral > neutral	3	Talairach	BrainVoyager 1999 version 3.9	1.5
Parkinson et al. (2011)	38 (NA)	NA	NA	Scenarios	All moral stories > neutral	1	Talairach	AFNI	3.0
Prehn et al. (2008)	23 (23)	25.17 (6.56)	NA	Statements	Socio‐normative > grammatical judgment	6	MNI	FEAT	1.5
Robertson et al. (2007)	16 (0)	35 (5.1)	27‐42	Scenarios	Moral (care/justice) > neutral	5	Talairach	SPM2	3.0
Schaich Borg et al. (2008)	50 (0)	25 (6)	NA	Statements	Sociomoral (incest and nonsexual moral) > neutral and pathogen	17	MNI	SPM2	3.0
Sevinc et al. (2017)	22 (10)	22.1 (3.14)	NA	Scenarios	Moral and immoral > neutral	24	MNI	NA	3.0
Smith et al. (2015)	30 (25)	26 (NA)	19‐36	Scenarios	Equal and unequal > control	7	MNI	SPM8	1.5
Sommer et al. (2010)	12 (7)	24.5 (2.1)	NA	Scenarios	Moral conflicts > neutral conflicts	6	MNI	SPM5	3.0
Sommer et al. (2014)	16 (8)	24.9 (2.96)	22‐31	Scenarios	Moral conflicts > social conflicts	6	MNI	SPM5	3.0
Takahashi et al. (2008)	15 (NA)	20.1 (0.8)	NA	Statements	Moral beauty > neutral	5	MNI	SPM2	1.5
Tsoi et al. (2018)	25 (7)	28.56 (10.1)	18‐50	Scenarios	Psychological > neutral	22	MNI	SPM8	3.0
					Physical > neutral	3			
Wang et al. (2015)	28 (14)	22 (NA)	18‐25	Scenarios	Scene moral aesthetic judgment (moral vs. neutral) > gender judgment (male vs. female)	10	MNI	SPM8	3.0
Wen et al. (2020)	18 (9)	20.6 (NA)	19‐25	Scenarios	(MCPL‐MCPH) > (SCPL‐SCPH)	11	MNI	SPM8	3.0
					(MCSL‐MCSH) > (SCSL‐SCSH)	10			
Yoder and Decety (2014)	40 (21)	21.0 (2.58)	NA	Scenarios	Morally good > morally bad behavior	15	MNI	SPM8	3.0

***Note***: n, contrast number; *N*, sample size; S1, Study 1; Exp., experiment; NA, not available; *SD*, standard deviation; MNI, Montreal Neurology Institute.

**TABLE 2:** Summary of studies included in the meta‐analysis of dilemma moral tasks (*n* = 30).

Article	*N* (female)	Mean age (*SD*)	Age range	Stimuli	Contrast	Foci	SPACE	SOFT	Tesla
Cecchetto et al. (2019)	28 (28)	23.7 (4.2)	19‐34	Scenarios	Incongruent > congruent dilemmas	7	MNI	SPM12	3.0
Chiong et al. (2013)	16 (10)	66 (5.5)	NA	Scenarios	Moral personal > non‐moral dilemma	3	MNI	SPM5	3.0
					Moral personal > moral impersonal dilemma	4			
Cui et al. (2021)	33 (15)	22.24 (3.73)	NA	Statements	High level of moral conflict > low level of moral conflict	27	MNI	SPM12	3.0
de Achával et al. (2013), Exp.2	13 (5)	28.5 (8.6)	NA	Scenarios	Moral dilemma > non‐moral dilemma	6	Talairach	SPM8	3.0
FeldmanHall et al. (2014)	35 (22)	24.6 (3.8)	NA	Scenarios	Difficult moral > difficult non‐moral	6	MNI	SPM5	3.0
					Easy moral > easy non‐moral	4			
Greene et al. (2001), S1	9 (4)	NA	NA	Scenarios	Moral personal > moral impersonal/non‐moral	5	Talairach	NA	3.0
Greene et al. (2004)	38 (17)	NA	NA	Scenarios	Personal > impersonal moral judgment	14	Talairach	NA	3.0
					Difficult > easy personal moral judgments	9			
Han et al. (2014)	16 (8)	28.59 (3.18)	21‐34	Scenarios	Moral personal > neutral (Korean vs. American)	2	MNI	SPM8	3.0
					Moral impersonal > neutral (Korean vs. American)	1			
					Moral personal > impersonal (American vs. Korean)	1			
Han et al. (2016)	16 (8)	28.59 (3.18)	21‐34	Scenarios	Moral personal > control	11	MNI	SPM8	3.0
					Moral impersonal > control	14			
					Personal > impersonal	1			
Harrison et al. (2012)	73 (31)	32.6 (10.3)	19‐61	Scenarios	Moral personal > non‐dilemma	11	MNI	SPM5	1.5
Lim et al. (2017)	19 (10)	22.63 (1.3)	NA	Scenarios	Moral dilemma > non‐moral	30	MNI	FEAT	3.0
Ngo et al. (2019)	18 (0)	27.4 (6.3)	20‐41	Scenarios	Moral dilemma > neutral dilemma	25	MNI	SPM8	3.0
Pujol et al. (2012)	22 (0)	40.6 (9.5)	28‐61	Scenarios	Moral dilemma > control	14	MNI	SPM5	1.5
Preckel et al. (2015), Exp 1	23 (0)	24.13 (4.48)	NA	Scenarios	Moral dilemma > non‐moral	19	MNI	SPM8	1.5
Reniers et al. (2012)	24 (0)	N/A	18‐30	Scenarios	Moral > non‐moral dilemma	6	Talairach	SPM5	3.0
Schaich Borg et al. (2006)	24 (11)	22.9 (NA)	18‐32	Scenarios	Moral dilemma > non‐moral	7	MNI	SPM99	1.5
Schleim et al. (2011)	40 (18)	31.05 (4.02)	NA	Scenarios	Moral dilemma > neutral	6	Talairach	BrainVoyager QX 1.9	3.0
Schneider et al. (2013)	28 (NA)	31.29 (9.03)	18‐55	Scenarios	Moral dilemma > non‐moral	4	MNI	SPM8	3.0
Shenhav and Greene (2014)	35 (19)	22.9 (NA)	18‐32	Scenarios	Integrative moral judgment > emotional	4	MNI	SPM8	3.0
Sharvit et al. (2020), Exp2.	27 (14)	24.22 (4.23)	18‐33	Scenarios	Moral > non‐moral dilemmas (reading epochs)	6	MNI	SPM12	3.0
					Moral > non‐moral dilemmas (rating epochs)	2			
van Gils et al. (2020)	17 (17)	NA	NA	Scenarios	Sacrifice out‐group > in‐group	1	Talairach	Brain Voyager 21.4	3.0
Verdejo‐Garcia et al. (2014)	14 (1)	30.1 (8.8)	18‐50	Scenarios	Moral dilemma > control	8	MNI	SPM8	1.5

*Note*: n, contrast number; *N*, sample size; S1, Study 1; Exp., experiment; NA, not available; *SD*, standard deviation; MNI, Montreal Neurology Institute.

Formal Institutional Review Board (IRB) approval was not required because the study uses anonymized, publicly available data from previously published trials.

#### 2.1.2 Activation Likelihood Estimation (ALE) Analysis

A coordinate‐based meta‐analysis of reported fMRI experiments was performed using the GingerALE version 2.3.6 software package (http://brainmap.org) (Eickhoff et al. 2009). Employing the ALE algorithm, the reported coordinates of brain areas associated with dilemma and non‐dilemma moral tasks were separately assessed for convergence across experiments. ALE assesses the convergence of foci reported across fMRI experiments in a standardized stereotaxic space such as Talairach or MNI (Laird et al. 2005; Turkeltaub et al. 2002). ALE interprets reported foci as 3D Gaussian spatial probability distributions, whose widths are based on empirical estimates of the spatial uncertainty due to between‐subject and between‐template variability of the neuroimaging data (Eickhoff et al. 2009). Within each included study, a modulated activation (MA) map was first created by taking the maximum probability associated with any one focus (always the closest one) for each voxel (Turkeltaub et al. 2012). The union of these individual MA maps was then calculated to obtain an ALE map across studies. This ALE map was assessed against a null distribution of random spatial association between experiments using a nonlinear histogram integration algorithm (Eickhoff et al. 2012). All ALE maps were thresholded using a cluster‐level family‐wise error (cFWE) correction at *p* < 0.05, with a cluster‐forming threshold of *p* < 0.001 and 10,000 permutations to correct for multiple comparisons (Eickhoff et al. 2012, 2016). In ALE, cluster‐level correction is preferred over voxel‐level FWE correction. The voxel‐wise correction considers each voxel as a statistical analysis, whereas neural activation patterns typically span multiple voxels, which may lead to missing true effects. In contrast, cluster‐wise correction first identifies clusters of contiguous voxels that are activated above a certain threshold. It then controls the probability of observing at least one such cluster by chance, given the spatial characteristics of the data. Compared to the voxel‐wise correction, the cluster‐wise correction is less conservative, while still controlling the overall false positive rate. Cluster‐level FWE correction is currently regarded as the standard approach for ALE meta‐analysis (Eickhoff et al. 2012, 2016). ALE analyses were also conducted for different content valences of non‐dilemma moral tasks, but they were not performed on dilemma tasks because all content in the dilemma tasks had negative valence.

#### 2.1.3 Conjunction and Contrast Analyses

To identify brain regions that were common or unique to dilemma and non‐dilemma moral tasks, we further performed a conjunction analysis and two contrast analyses (i.e., non‐dilemma vs. dilemma moral tasks, dilemma vs. non‐dilemma moral tasks). We first merged all the original activation coordinates in dilemma and non‐dilemma moral tasks, and then conducted an individual meta‐analysis based on these pooled coordinates. The conjunction analysis was implemented using the minimum conjunction of ALE results of dilemma and non‐dilemma moral tasks using cFWE correction at *p* < 0.05 and a voxelwise cluster‐forming threshold of *p* < 0.001 (Nichols et al. 2005).

Next, contrast analyses were conducted to identify the statistically significant differences in the convergence between dilemma and non‐dilemma moral tasks. Contrast analyses were based on voxel‐wise differences of ALE maps of dilemma and non‐dilemma moral tasks (Eickhoff et al. 2011). To test statistical significance, permutation tests were conducted as follows. We pooled all experiments, randomly reassigned them to two groups matching the original sample sizes, and repeated the analysis under the assumption of label exchangeability (Gu et al. 2019; Langner et al. 2018). Based on each new data set, an ALE image was created for each group, and the two images were subtracted and compared with the original data. After 10,000 permutations, we generated a null distribution of ALE differences between the two task conditions and computed voxel‐wise *p*‐values (Laird et al. 2005). Finally, we applied an uncorrected threshold of *p* < 0.05 with a minimum cluster size of 100 mm^3^ to correct for false positive inflation resulting from multiple comparisons. All results are represented in the MNI space.

Additionally, we conducted conjunction and contrast analyses for dilemma type (statements vs. scenarios), perspective (first‐person vs. third‐person), and different content valences of non‐dilemma moral tasks (e.g., negative ∩/> positive valence, negative ∩/> mixed valence) and the negative valences of both dilemma and non‐dilemma moral tasks.

Moreover, to assess the robustness of the ALE findings against potential publication bias, we conducted a Fail‐Safe N (FSN) analysis following Acar et al. (2018). FSN estimates the number of simulated null (“noise”) experiments that can be added before a significant ALE cluster becomes non‐significant. For each significant cluster, a lower boundary was first calculated as 30% of the total number of experiments, based on estimates of potential publication bias in neuroimaging research (Samartsidis et al. 2019). The upper boundary was calculated as ((*C*/0.10) − *K*), where (*C*) denotes the number of experiments contributing to the cluster and (*K*) denotes the total number of experiments in the original ALE analysis (Arioli et al. 2026). Noise experiments were generated in R (version 3.6.1) using the code and procedures of Acar et al. (2018). The simulated experiments matched the empirical studies in sample size and number of reported foci, and the foci were randomly distributed across the brain. The noise experiments were then incrementally added to the original dataset, and the ALE analysis was repeated using the same parameters as the original analysis (voxel‐level *p* < 0.001, cluster‐level FWE‐corrected *p* < 0.05, and 10,000 permutations). The FSN was determined iteratively by identifying the number of added noise experiments at which the previously significant cluster became non‐significant, with the search progressively narrowed between the lower and upper boundaries. A larger FSN indicates greater robustness of the ALE finding against potential publication bias.

### 2.2 Meta‐analytical Connectivity Modeling (MACM) Analyses

The MACM approach enables the evaluation of co‐activation patterns across numerous neuroimaging studies and facilitates the identification of predefined regions of interest (ROIs) based on functional connectivity maps (Langner et al. 2014). We identified clusters from the contrast analyses (i.e., dilemma > non‐dilemma moral tasks, non‐dilemma > dilemma moral tasks) and used these as ROIs in the BrainMap database (http://www.brainmap.org/). Each ROI served as a seed to find studies reporting activations within the cluster (Laird et al. 2009). Our analysis included only whole‐brain neuroimaging studies with activations reported in standard stereotaxic space for healthy participants. For clusters from the contrast of non‐dilemma versus dilemma moral tasks, there were 165 experiments (2313 foci, 2863 participants) reporting activation in the right vmPFC, 99 experiments (1322 foci, 1551 participants) reporting activation in the left OFC, 35 experiments (456 foci, 565 participants) reporting activation in the right TP, and 191 experiments (2589 foci, 3264 participants) reporting activation in the right precuneus. In the contrast between dilemma and non‐dilemma moral tasks, 248 experiments (3152 foci, 4113 participants) reported activation in the left dmPFC (dorsomedial prefrontal cortex), 300 experiments (3715 foci, 5013 participants) in the left TPJ, and 322 experiments (3901 foci, 5323 participants) reported activation in the right TPJ. The coordinates from the retrieved experiments were subsequently analyzed using the ALE algorithm to detect areas of convergent co‐activation for each seed. Statistical significance was set at *p* < 0.05, corrected for multiple comparisons.

### 2.3 Network‐Based Meta‐Analyses

We used the ANM method to identify brain network patterns associated with dilemma and non‐dilemma moral tasks. This method was adapted from lesion network mapping (LNM), a widely employed technique that identifies symptom‐related brain networks in patients with brain lesions (Peng et al. 2022). First, we created an 8‐mm radius sphere centered on each of the reported peak voxels (i.e., x, y, z coordinates) from the experiments included in the present meta‐analysis. Next, for each experiment, we created a combined activation seed by merging the coordinate‐level spheres from the same experiment. Because each experiment had one combined activation seed, there were 22 combined activation seeds for dilemma and 30 for non‐dilemma moral tasks. Such combined activation seeds allowed us to draw experiment‐level random‐effects inferences from the subsequent analyses (Eickhoff et al. 2009; Yarkoni et al. 2011).

Second, we performed whole‐brain resting‐state functional connectivity analyses (RSFC) using the combined activation seeds as ROIs. This analysis identified activation network maps for the ROI seeds for each contrast type, i.e., brain regions that were functionally connected to the ROI seed regions. The RSFC was derived from newly acquired resting‑state fMRI data in a sample of 370 healthy college students. Following the exclusion of 4 participants with excessive head motion (>3 mm translation or 3° rotation), 366 participants (119 females; 24.4 ± 4.99 years) were included in the final analysis. We obtained the 8‐min resting‐state functional scans using a 3T Siemens Magnetom Prisma Scanner at the Life Science Imaging Center, Hong Kong Baptist University, as part of a larger fMRI project. This project (REC/22‐23/0503) was approved by the Hong Kong Baptist University Institutional Review Board, and all participants were provided written informed consent. The data are not currently publicly available. During the resting‐state fMRI scans, participants were instructed to fixate on a cross positioned at the center of the screen. The functional images were acquired with an echo‐planar imaging (EPI) sequence (60 slices, slice thickness = 2.2 mm, TR = 1000 ms, TE = 29 ms, FA = 45°, FOV = 260 mm, band acceleration factor = 4, voxel size = 2.5 × 2.5 × 2.0 mm^3^). For registration, we acquired T1‐weighted images (192 slices, slice thickness = 1 mm, TR = 2300 ms, TE = 2.28 ms, FA = 8°, FOV = 256 mm, Voxel Size = 1.0 × 1.0 × 1.0 mm^3^). Preprocessing was conducted using Statistical Parametric Mapping (SPM12). To achieve a steady state in neuroimaging, we discarded the first 10 images from the analysis to mitigate non‐equilibrium magnetization effects. EPI data were then corrected for slice‐timing differences and realigned to the first scan to address head motion. Several nuisance variables, including six head motion parameters, white matter, cerebrospinal fluid, and global signals, were regressed out. Additionally, all images underwent temporal bandpass filtering (0.01–0.1 Hz) and spatial smoothing at 6 mm FWHM. For each participant, the RSFC analysis produced an *r* map representing Pearson's correlation coefficient between the average BOLD signals of all voxels within the ROI seed and the signal at every voxel in the whole brain. We then transformed the subject‐level *r* maps to Fisher *z* maps via Fisher's *z* transformation.

Third, to identify the activation network maps for each contrast type, we adopted the overlap‐based analytic approach. We compared Fisher *z* maps for each experiment‐level ROI seed against zero using voxel‐wise one‐sample *t*‐tests thresholded at voxel‐level *p* < 0.001. We then thresholded and binarized the resulting experiment‐level *t*‐maps belonging to the same contrast type at *t* > 5.23 (equivalent to voxel‐wise FWE‐corrected *p* < 0.05). The binarized maps from the same contrast type were overlapped and thresholded at 90% (i.e., overlap in the activation network maps of more than 90% of the experiment‐level ROI seeds) to create activation network overlap maps for each contrast type. It is worth noting that the “percentage” threshold used to define group‐level overlap maps has been arbitrary and inconsistent across previous studies, ranging from 60% to over 90%. The power of ANM lies not in claiming that a thresholded network is statistically “significant,” but in demonstrating that the highest‐ranked voxels within the network are consistently aligned across studies and that the ranked network can be validated through its overlap with evidence from other modalities, such as TMS or lesion studies. This ranking provides a stable and meaningful signature of the underlying cognitive process, regardless of the specific threshold used for visualization.

### 2.4 Meta‐Analytical Functional Decoding

Given the challenges of reverse inference, which involves deducing cognitive functions from observed brain activity (Poldrack 2006), Neurosynth Image Decoder (https://neurosynth.org/decode/) was used to decode the functional characteristics of each meta‐analysis network. This platform computes Pearson's correlation coefficient across all voxels to compare any meta‐analysis network to other term‐specific meta‐analytic maps (Bellucci et al. 2019). For dilemma and non‐dilemma moral tasks, and their conjunction, we listed the top 20 ranked terms per map and selected four relevant meta‐analytical activation maps based on the size of voxel‐wise Pearson correlation coefficients (Tan et al. 2022) for the contrast and conjunction analyses (i.e., dilemma > non‐dilemma moral tasks: evaluation, theory mind, beliefs, and mind; non‐dilemma > dilemma moral tasks: social cognitive, communication, comprehension, and theory mind; and dilemma ∩ non‐dilemma moral tasks: theory mind, mental states, mentalizing, and moral).

Furthermore, we performed functional characterization using the behavioral domain metadata categories that were available for each neuroimaging experiment in the BrainMap database (http://www.brainmap.org/scribe/). According to these metadata categories, each study in the database classified its experimental properties into five major behavioral domains (action, cognition, emotion, perception, and interoception) with 51 sub‐domains (Fox et al. 2005; Turner and Laird 2012). BrainMap behavioral profiling analyses were performed on each region identified in the conjunction analysis and on each subcluster that was identified from the contrast analysis using the Behavioral analysis plugin software (https://mangoviewer.com/plugin_behavioralanalysis.html) in the BrainMap database, and Lancaster et al. (2012) provide further details of the procedure. The BrainMap behavioral profiling helps researchers identify regionally specific behaviors for their brain studies, and only behavioral sub‐domains with positive z‐scores ≥ 3.0 were considered significant (*p* ≤ 0.05 with Bonferroni correction for multiple comparisons); a behavior profile chart and z‐score‐ranked table or figure were provided to aid interpretation (Lancaster et al. 2012).

Additionally, to further examine whether the activity of a region was more consistent in studies mentioning the current term than in studies that do not, we performed term‐based meta‐analyses with association and uniformity tests using “moral” as a term in Neurosynth (https://neurosynth.org/analyses/terms/). The association test is similar to reverse inference, estimating the probability of a term given observed activation [P(Term|Activation)]. In contrast, the uniformity test aligns with forward inference, reflecting the probability of activation given the presence of a term [P(Activation|Term)] (Yarkoni et al. 2011).

## 3 Results

### 3.1 Individual Meta‐Analysis

The ALE analyses revealed distinct neural patterns for non‐dilemma and dilemma moral tasks. Non‐dilemma processing consistently engaged a network comprising the left dmPFC, right vmPFC, left precuneus, left TPJ/STG (superior temporal gyrus), right TP, left anterior cingulate cortex (ACC), and left OFC (Figure 2A and Table 3a). In contrast, dilemma moral processing preferentially activated the left dmPFC, left precuneus, and bilateral TPJ/STG (Figure 2B and Table 3b).

**FIGURE 2:**
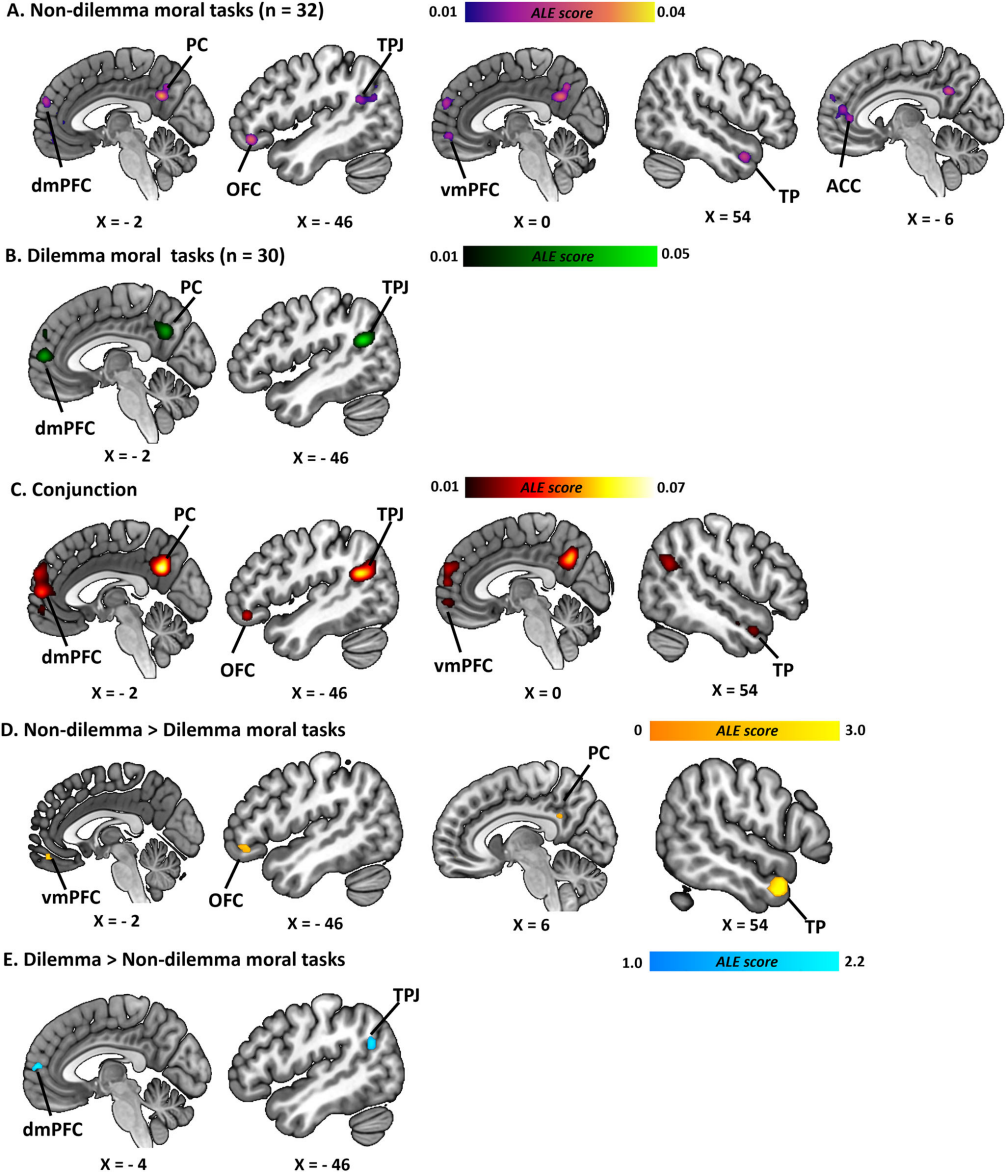
Significant clusters from the main coordinate‐based ALE meta‐analysis (cluster‐level family‐wise error correction [*p* < 0.05] with a cluster‐forming threshold of *p* < 0.001 using 10,000 permutations) for non‐dilemma versus control, dilemma versus control, their conjunction analyses, and contrasts. Consistent activation clusters for (A) non‐dilemma versus control; (B) dilemma versus control; (C) conjunction analysis between non‐dilemma and dilemma tasks; (D) contrast analyses between non‐dilemma and dilemma tasks; (E) contrast analyses between dilemma and non‐dilemma tasks. *n*, number of contrasts; TP, temporal pole; ACC, anterior cingulate cortex; OFC, orbitofrontal cortex; PC, precuneus; vmPFC, ventromedial prefrontal cortex; dmPFC, dorsomedial prefrontal cortex; TPJ, temporoparietal junction.

**TABLE 3:** ALE meta‐analysis results on non‐dilemma tasks, dilemma tasks, their conjunctions, and contrasts.

Cluster *#*	Brain regions	BA	MNI coordinate (mm)			Peak *Z* score	Cluster size (voxels)
			x	y	z		
***a. Non‐dilemma tasks***							
1	Precuneus (L)	31	−2	−54	30	6.27	424
2	TPJ/STG (L)	22	−48	−54	22	4.36	339
3	TPJ/STG (R)	39	58	−60	24	4.24	87
4	TP (R)	21	54	6	−26	5.58	205
5	dmPFC (L)	9	2	58	24	4.22	146
6	ACC (L)	32	−6	42	4	4.27	142
7	vmPFC (R)	/	4	56	−8	4.00	104
8	OFC (L)	47	−46	38	−12	5.54	140
***b. Dilemma tasks***							
1	Precuneus (L)	31	0	−58	34	7.61	424
2	dmPFC (L)	9	−4	54	10	6.28	379
3	dmPFC (L)	6	−4	54	32	4.20	152
4	TPJ/STG (R)	39	52	−58	26	4.94	438
5	TPJ/STG (L)	39	−46	−58	24	7.17	329
***c. Conjunction***							
1	dmPFC (L)	9	−4	54	10	6.77	1056
2	TPJ/STG (L)	39	−48	−58	24	7.75	641
3	TPJ/STG (R)	39	58	−58	22	6.14	526
4	Precuneus (L)	31	0	−56	32	8.97	697
5	TP (R)	21	54	6	−26	4.92	201
6	vmPFC (R)	10	4	60	−8	4.23	151
7	OFC (L)	/	−28	18	−12	4.19	146
8	OFC (L)	47	−46	38	−12	5.40	101
***d. Non‐dilemma > Dilemma***							
1	TP (R)	21	55	8	−28	3.04	192
2	OFC (L)	47	−48	36	−8	2.49	79
3	vmPFC (L)	10	−2	48	−16	2.48	30
4	Precuneus (R)	31	6	−44	30	2.05	18
***e. Dilemma > Non‐dilemma***							
1	dmPFC (L)	9	−4	58	12	2.17	36
2	TPJ/STG (L)	39	−46	−60	26	2.12	36
3	TPJ/STG (R)	39	48	−54	30	1.97	22
4	TPJ/STG (R)	13	52	−46	18	1.90	15

*Note*: L, left hemisphere; R, right hemisphere; BA, Brodmann area; MNI, Montreal Neurological Institute; vmPFC, ventromedial prefrontal cortex; dmPFC, dorsomedial prefrontal cortex; OFC, orbital frontal cortex; TPJ, temporoparietal junction; STG, superior temporal gyrus; TP, temporal pole; ACC, anterior cingulate cortex.

Valence‐based sub‐analyses indicated that negative‐valence content elicited activation in the left dmPFC, left vmPFC, left precuneus, and right TPJ in non‐dilemma moral tasks (Table S5A). No significant clusters were identified for positive‐valence content (Table S5B), whereas mixed‐valence content engaged the left OFC, left TPJ, and right STG in non‐dilemma moral tasks (Table S5C).

The scenario‑based sub‑analyses revealed that, in moral dilemma tasks, scenario‑induced dilemmas engaged the left dmPFC, left precuneus, bilateral TPJ, and right MTG (Table S6A), whereas non‑dilemma moral tasks recruited only the right precuneus (Table S6B). In contrast, when dilemmas were presented as statements rather than scenarios, non‑dilemma moral tasks activated the right vmPFC, left dmPFC, left precuneus, left TPJ, left OFC, and right MTG (Table S6C).

The perspective‑based sub‑analyses further showed that, for moral dilemma tasks, a first‑person perspective elicited activation in bilateral TPJ, left precuneus, and left dmPFC (Table S7A), while for non‑dilemma moral tasks, it involved only the right TPJ and left precuneus (Table S7D). A third‑person perspective, in the context of non‑dilemma moral tasks, activated the right vmPFC, left precuneus, left TPJ, left OFC, and right TP (Table S7C).

FSN analyses were conducted for the dilemma and non‐dilemma moral task ALE maps. The resulting FSN values substantially exceeded the corresponding lower boundaries. Thus, the significant ALE findings for both task types showed considerable robustness to potential publication bias (Table S13).

### 3.2 Conjunction and Contrast Analysis

Conjunction analysis identified common activation across both task types in regions including the left dmPFC, right vmPFC, bilateral TPJ/STG, left precuneus, left OFC, and right TP (Figure 2C and Table 3c), suggesting a shared neural substrate for dilemma and non‐dilemma moral processing. Valence‐specific conjunction results are detailed in Supplementary Materials Table S5. Notably, the conjunction between negative‐valence dilemma and non‐dilemma tasks revealed overlap in the left dmPFC, left precuneus, and bilateral TPJ (Table S5K), underscoring the role of these regions in processing affectively salient moral content. Further, dilemma status conjunctions showed that scenario‑based dilemmas commonly engaged the left dmPFC, left precuneus, bilateral TPJ, and bilateral insula across both task types (Table S6D). In terms of question perspective, the first‑person conjunction commonly activated the left dmPFC, left precuneus, bilateral TPJ, left caudate, left putamen, and right insula (Table S7E), whereas the cross‑perspective conjunction—contrasting first‑person in dilemma tasks with third‑person in non‑dilemma tasks—revealed overlap in the left dmPFC, right vmPFC, bilateral TPJ, left precuneus, left OFC, and right MTG (Table S7F).

Contrast analyses demonstrated that non‐dilemma tasks preferentially engaged the left vmPFC, left OFC, right precuneus, and right TP (Figure 2D and Table 3d), regions associated with affective evaluation, social inference, and self‐referential processing. Dilemma moral tasks more strongly recruited the bilateral TPJ/STG and left dmPFC (Figure 2E and Table 3e), implicating mentalizing and conflict monitoring. Compared with dilemma moral tasks, non‑dilemma moral tasks involving negative‑valence content elicited greater activation in the right precuneus and left ACC (Table S5L), whereas no regions showed significantly greater activation in the opposite contrast (Table S5M). For scenario‑based dilemmas, the dilemma condition induced significantly greater activation in the right dmPFC and right TPJ than did the non‑dilemma condition (Table S6D), while the non‑dilemma condition engaged the left OFC more strongly than did the dilemma condition (Table S6E). When dilemmas were presented as statements rather than scenarios, the non‑dilemma condition produced significantly greater activation in the left dmPFC, left vmPFC, left TPJ, and left OFC (Table S6F); no significant regions were observed for the opposite comparison (scenario > statement) within the non‑dilemma context (Table S6G). Regarding the perspective of questions, the first‑person perspective in non‑dilemma moral tasks elicited greater activation in the left precuneus and bilateral TPJ compared with the dilemma condition (Table S7H). In contrast, the first‑person perspective in dilemma tasks evoked stronger activation in the left dmPFC, right TPJ, right precuneus, and left MTG relative to the third‑person perspective in non‑dilemma tasks (Table S7I). Conversely, the third‑person perspective in non‑dilemma tasks showed greater activation in the left vmPFC, left OFC, and right TP compared with the first‑person perspective in dilemma tasks (Table S7J).

To rule out potential bias from participant overlap and multiple contrasts within the same study, we performed sensitivity analyses by combining related contrasts and retaining only one contrast per sample when studies shared the same participants (see Tables S8 and S9). The results were highly consistent with the original ALE, conjunction, and contrast findings (see Table S10), confirming that our findings are robust and unlikely to be driven by sample overlap or non‑independent contrasts.

### 3.3 MACM

MACM results indicated that during non‐dilemma moral tasks, key regions—including the amygdala, ACC, and vmPFC—exhibited co‐activation patterns aligning with the salience network (SN), DMN, and limbic system (Table S11A/B/C and Figure S1A). For dilemma tasks, functional connectivity involved the amygdala, parahippocampus, PCC, vmPFC, and dorsolateral prefrontal cortex (dlPFC), highlighting contributions from the DMN, SN, central executive network, and limbic regions (Table S11D,E,F,G and Figure S1B). These findings reflect divergent large‐scale network engagement between non‐dilemma and dilemma tasks.

### 3.4 ANM

Overlap analysis confirmed that both non‐dilemma and dilemma tasks showed convergent connectivity patterns involving regions commonly implicated in the mesocorticolimbic system, including the bilateral striatum and mPFC (Figure S2), suggesting a common neurofunctional foundation for moral decision‐making rooted in reward and self‐related processing.

It has been argued that the network patterns identified using LNM and its variants, such as ANM, may primarily reflect the hub architecture of the normative connectome rather than condition‐specific circuitry (van den Heuvel et al. 2026). However, recent research has shown that ANM does not consistently yield similar network patterns across conditions (Cui et al. 2026). For example, our research identified markedly different networks associated with regret and relief (Varma et al. 2023). ANM also revealed little overlap between temporal and effort discounting (Varma et al. 2023). If ANM primarily identifies connectivity associated with hub regions, we would expect substantial overlap across moral and non‐moral conditions. Taken together with published evidence, the network derived from the current study is unique and meaningful rather than a generic “hub” artifact.

### 3.5 Meta‐Analytical Functional Decoding

Functional decoding via meta‐analytic network mapping revealed that processing moral dilemmas was associated with value evaluation and mental‐state attribution, whereas non‐dilemma processing was linked more strongly to social cognition and communication. Both tasks involved mentalizing, mental states, and morality‐related processes (Figure S3A and Table S12).

Behavioral domain analysis in BrainMap confirmed that both task types correlated with social cognition in the left TPJ (Figure S3B and Table S14A). Moral dilemma processing was linked to action execution in the left TPJ (Figure S4A and Table S14B), whereas non‐dilemma processing was associated with working memory in the right TP (Figure S4B and Table S14C).

The association test identified convergence in moral decision‐making processing within the right dmPFC, right vmPFC, right OFC, bilateral TPJ, and right precuneus (Table S15A), corroborating conjunction results. The uniformity test further indicated that moral decision‐making broadly recruits the left dmPFC, right precuneus, bilateral insula, bilateral TPJ, dlPFC, left inferior frontal gyrus (IFG), left parahippocampus, right amygdala, and left MTG (Table S15B), reflecting a distributed yet integrated neural system.

## 4 Discussion

The present meta‐analysis aimed to elucidate the common and distinct neural substrates underlying dilemma and non‐dilemma moral tasks. Convergent results from ALE, MACM, and functional decoding revealed that although both types of moral tasks engage a shared core network—including the left dmPFC, right vmPFC, bilateral TPJ, left precuneus, left OFC, and right TP—they also recruit dissociable neural systems reflecting their differential cognitive and affective demands. Specifically, the left vmPFC, left OFC, right precuneus, and right TP were more consistently engaged during non‐dilemma processing, whereas the bilateral TPJ and left dmPFC showed stronger activation during dilemma processing.

### 4.1 Brain Regions Showing Greater Involvement in the Non‐Dilemma Tasks

Results revealed stronger convergence in the left vmPFC, left OFC, right precuneus, and right TP during non‐dilemma tasks. Accumulated evidence indicates that the vmPFC plays a critical role in moral decision‐making (Blair et al. 2006; Fumagalli and Priori 2012; Marazziti et al. 2013; Raine and Yang 2006). For example, patients with vmPFC lesions exhibit impaired social behavior—such as irresponsibility and inappropriate conduct—despite retaining normal cognitive function and explicit knowledge of moral norms (Anderson et al. 1999; Saver and Damasio 1991), underscoring the vmPFC's essential role in evaluating social norms and values. Moreover, the vmPFC is involved in representing and computing subjective value (Bartra et al. 2013; Qu et al. 2020; Qu et al. 2019) and facilitates moral learning (Qu et al. 2022). A meta‐analysis identified the vmPFC and ventral striatum as core components of the brain's valuation system, which supports value‐based decision‐making (Bartra et al. 2013). Notably, vmPFC activity reflects sensitivity to both positive and negative valences when evaluating anticipated outcomes in moral contexts, such as when the severity of moral violations is manipulated (Luo et al. 2006). Additionally, the vmPFC contributes to the generation of social emotions—including shame, guilt, and compassion—particularly in situations involving violations of social norms (Koenigs and Tranel 2007; Koenigs et al. 2007). Together, these findings suggest that the vmPFC contributes to representing moral values and generating social emotions.

The OFC has been consistently implicated in representing the expected value of behavioral outcomes (Amodio and Frith 2006; Camille et al. 2004; Luo et al. 2006; Walton et al. 2004) and in guiding context‐appropriate behavior selection (Rudebeck and Murray 2014). It shows robust activation during the encoding of subjective value for chosen options (Padoa‐Schioppa and Assad 2006) and participates directly in decision formation (Rich and Wallis 2016). Evidence from animal studies further indicates that OFC stimulation disrupts value computation and comparison (Ballesta et al. 2020), underscoring its role in linking choices to value signals. Additionally, OFC neurons can rapidly update outcome expectations based on incomplete information about changing task contingencies (Stalnaker et al. 2014), highlighting its capacity to represent and revise task states—especially under abstract or perceptually ambiguous conditions (Takahashi et al. 2011). Collectively, these findings support the view that the OFC may be associated with the computation and continuous updating of moral values.

The precuneus plays a multifaceted role in social cognition (Cavanna and Trimble 2006), self‐referential processing (Young et al. 2007), and egocentric spatial awareness (Freton et al. 2014). It exhibits stronger activation in response to self‐relevant than non‐self‐relevant information (Kircher et al. 2000), and is specifically engaged when processing intentions linked to the self (den Ouden et al. 2005). As a core node of the DMN (Buckholtz and Marois 2012; Fox et al. 2005), the precuneus also contributes to autobiographical memory retrieval (Andrews‐Hanna et al. 2014) and mental simulation (Spreng and Grady 2010). These functions collectively suggest that the observed activation of the precuneus during non‐dilemma moral tasks may reflect processes related to self‐referential and personally relevant information integration, likely by facilitating access to autobiographical memories when evaluating clear moral transgressions. Specifically, within the negative context of non‐dilemma moral tasks involving more negative emotion, activation of the precuneus may indicate that individuals evaluate moral rationality by engaging self‐referential processing, using their own perspectives and values as a framework for evaluation.

The TP has been consistently linked to the processing of personal and episodic memories, as evidenced by its activation in response to visually presented autobiographical material (Gorno‐Tempini et al. 1998; Markowitsch 1995; Nakamura et al. 2000; Nakamura and Kubota 1996) and during the comprehension of self‐referential sentences (Fink et al. 1996). Lesion studies further support its functional importance; damage to the right TP is associated with impaired recognition of familiar faces, landmarks, and scenes (Ellis et al. 1989; Markowitsch et al. 1993; Nakamura and Kubota 1996), suggesting a critical role in long‐term memory retrieval and the storage of socially relevant knowledge. Our functional decoding results align with these findings, indicating that non‐dilemma moral processing engages neural networks subserving social norms, language comprehension, and working memory. This supports the view that the vmPFC, OFC, TP, and precuneus interact to compute moral values by integrating autobiographical memories and dynamically updating subjective valuations during such tasks. Thus, TP may contribute to moral cognition by providing access to past personal experiences that guide moral judgment.

### 4.2 Brain Regions Showing Greater Involvement in the Dilemma Moral Tasks

Converging evidence indicates that dilemma processing engages a network including the left dmPFC and bilateral TPJ. The left dmPFC, in particular, has been consistently linked to conflict processing (Stevens et al. 2009) and cognitive control (Shenhav et al. 2013). A recent meta‐analysis further highlighted its role in detecting and inhibiting conflict during contexts involving exploration and risk (Zhen et al. 2022). Single‐neuron recordings in macaques have shown that dmPFC cells encode species‐specific social rules (Wallis et al. 2001). Together, these findings suggest that the dmPFC contributes to dilemma‐based moral reasoning through its involvement in conflict detection, monitoring, and inhibitory control.

The TPJ is robustly implicated in mentalizing—the ability to attribute mental states to others (Döhnel et al. 2012; Frith and Frith 2003; Saxe et al. 2004; Schuwerk et al. 2014). It is consistently recruited during the processing of morally relevant information (Koster‐Hale et al. 2013; Young and Saxe 2009). Functional lateralization appears relevant, with the right TPJ particularly involved in representing moral norms, as evidenced by reduced activation in individuals with autism spectrum disorder (Hu et al. 2021), and in signaling conflicts between self‐interest and moral demands (Obeso et al. 2018; Qu et al. 2020, 2022; Strombach et al. 2015). Perturbation studies using TMS further confirm a causal role: disruption of the right TPJ reduces sensitivity to an agent's mental state and leads to more lenient moral judgments (Young et al. 2010), underscoring its importance in integrating intentionality cues during moral decision‐making (Cushman 2008; Killen et al. 2011; Knobe 2005). In negative scenarios of dilemma moral tasks, significant activation was also observed in the bilateral TPJ, underscoring its critical role in supporting mentalizing processes—such as inferring intentions and attributing mental states—under emotionally adverse moral conditions. Complementing these findings, behavioral decoding via BrainMap associated activity in the left TPJ with social cognition during moral dilemma processing. Together, this evidence suggests that the TPJ may contribute to spontaneous mental state inference in morally complex contexts, enabling individuals to navigate dilemmas involving competing social and personal interests (Carter and Huettel 2013; Young and Saxe 2009).

Our functional decoding results suggest that moral dilemma processing is associated with neural networks implicated in action execution, belief attribution, and mentalizing, a pattern consistent with the involvement of cognitive control, ToM, and self‐referential processing. Compared with non‐dilemma tasks, dilemma‐based tasks showed greater associations with processes related to conflict monitoring, mental‐state inference, and the retrieval of personally relevant beliefs. This is largely due to the inherent conflict in dilemmas, which requires individuals to simulate possible outcomes, draw upon deeply held values, and engage in deliberative reasoning (Greene et al. 2001, 2004). As such, moral dilemma processing may involve relatively slower and more controlled cognitive processing. In contrast, non‐dilemma processing may place greater reliance on the rapid evaluation of moral valence and the retrieval of affective and experiential representations, a pattern consistent with a relatively more automatic and emotion‐guided mode of moral judgment (Moll et al. 2002a).

### 4.3 Common Neural Activity of Non‐Dilemma and Dilemma Tasks

Both non‐dilemma and dilemma tasks consistently engaged a common set of brain regions, including the left dmPFC, right vmPFC, bilateral TPJ, left precuneus, left OFC, and right TP. These regions are associated with distinct yet complementary cognitive and affective functions: the dmPFC is related to cognitive control (Shenhav et al. 2013), the vmPFC is related to value representation (Bartra et al. 2013; Qu et al. 2020) and social emotions (Koenigs et al. 2007), the OFC may contribute to subjective valuation (Ballesta et al. 2020), the TPJ may be associated with mentalizing (Saxe et al. 2004), the precuneus is linked to self‐referential processing (Young et al. 2007), and the TP is related to the encoding of personal memories (Nakamura et al. 2000). These regions interact during moral cognition. The valuation (vmPFC/OFC) and mentalizing (dmPFC/TPJ) systems cooperate during social decision‐making (Hampton et al. 2008; Hill et al. 2017), particularly when self‐interest conflicts with moral demands (Qu et al. 2020). Their convergence across task types indicates a shared reliance on integrated cognitive–affective processes and supports interactive models of moral decision‐making (Greene and Haidt 2002), whereby judgments in both dilemmatic and non‐dilemmatic contexts emerge from dynamic coupling among valuation, mentalizing, self‐relevance, and affective systems. Our sub‐analysis results also provided evidence for this dynamic coupling perspective. Results showed that both negative‐valence dilemma and non‐dilemma tasks were related to mentalizing (dmPFC/TPJ) and self‐referential (precuneus) processing. Scenario‐based dilemma and non‐dilemma tasks also involved mentalizing, self‐referential, and affective (insula) processing. Moreover, first‐person questions in dilemma and non‐dilemma tasks also engaged mentalizing, self‐referential, affective, and reward (putamen/caudate) processing.

Moreover, our association test (similar to reverse inference) indicated that the identified brain regions, along with the TP, are strongly associated with the term “moral” in the Neurosynth database. Complementarily, a uniformity test (similar to forward inference) revealed that the dmPFC, TPJ, and precuneus, in particular, showed significant convergence with moral processing, suggesting that these regions are specifically correlated with moral cognition, though not all implicated areas are exclusively dedicated to it. BrainMap behavioral profiling further linked both task types to social cognition in the left TPJ. Functional decoding expanded these findings by revealing engagement of mentalizing, social cognition, belief attribution, and autobiographical memory across both non‐dilemma and dilemma tasks, which is also consistent with our main and sub‐conjunction analyses. Together, these findings highlight the contributions of self‐referential beliefs and personal memory, alongside valuation, conflict monitoring, and mental‐state inference, to moral decision making across different task contexts.

### 4.4 Brain Networks in Non‐Dilemma and Dilemma Tasks

MACM revealed both overlapping and distinct large‐scale networks underlying non‐dilemma and dilemma tasks. For non‐dilemma tasks, seed‐based analysis using the right vmPFC, left OFC, right precuneus, and right TP identified a coactivation network including the amygdala, ACC, and vmPFC. This pattern suggests that non‐dilemma processing engages integrated circuits supporting self‐reference, episodic memory, ToM, cognitive control, and emotional processing. For dilemma tasks, seeds in the left dmPFC and bilateral TPJ yielded a coactivation network involving the amygdala, parahippocampus, PCC, vmPFC, and dlPFC, indicating recruitment of networks related to self‐reference, episodic memory, ToM, emotion, working memory, reasoning, and planning. These results demonstrate that while both types of moral tasks engage shared processes—including self‐reference, episodic memory, ToM, cognitive control, and emotion—supporting the interplay between cognitive and affective processes in moral decision‐making (Greene and Haidt 2002), they also rely on distinct neural pathways: non‐dilemma tasks more strongly involve value‐related regions, whereas dilemma tasks additionally recruit cognitive control‐related networks.

Although prior work has highlighted interactions between valuation and mentalizing networks during social decision‐making (Hampton et al. 2008; Hill et al. 2017), particularly under moral conflict (Qu et al. 2020), our findings further specify that moral cognition flexibly engages both common and specialized neural systems depending on task demands. This adaptive mechanism allows individuals to modulate moral responses according to contextual requirements, ranging from rapid evaluative judgments to deliberative conflict resolution.

ANM further indicated that both dilemma and non‐dilemma tasks recruit the mesocorticolimbic network, which includes the bilateral striatum and mPFC. These findings align with computational models of moral decision‐making, proposing that individuals assess available options along a common value axis and select the option with the highest subjective value. This process initially involves encoding the subjective value of potential actions—relying on regions such as the striatum and anterior insula—followed by value integration and comparison, which engage the vmPFC and ACC (Ruff and Fehr 2014).

### 4.5 Comparison With Previous Meta‐Analyses

We contextualized our findings within recent meta‐analyses on moral decision‐making. Garrigan et al. (2017) directly compared moral response tasks and moral evaluation tasks. Notably, we identified significant activation in the vmPFC, OFC, precuneus, and TP during non‐dilemma tasks, whereas Garrigan et al. (2017) reported no significant activation for moral evaluation tasks relative to moral response tasks. This discrepancy may be attributable to our inclusion of a larger number of contrasts, enhancing statistical power to detect activation clusters.

Fede and Kiehl (2020) compared simple and utilitarian moral dilemma tasks. While our results implicate the TPJ and dmPFC in dilemma tasks, Fede and Kiehl (2020) did not observe significant activation in utilitarian moral dilemmas. Conversely, although we found the vmPFC, OFC, precuneus, and TP to be involved in non‐dilemma evaluation, Fede and Kiehl (2020) reported that simple moral dilemmas engaged the OFC, ACC, TPJ, amygdala, PCC, and insula. These inconsistencies may arise from methodological differences, including task classification approaches (criteria‐based vs. machine‐learning‐assisted), analytic algorithms (ALE vs. MKDA), and imbalances in the number of studies per condition (e.g., 32:30 in our study vs. 47:9 in theirs).

As noted earlier, Bzdok et al. (2012) primarily examined neural activity associated with moral cognition, ToM, and empathy. The definition of moral cognition in their study closely corresponds to the combined scope of non‐dilemma and dilemma tasks in our meta‐analysis. Consistent with their results, we also observed activation clusters in the vmPFC, dmPFC, TPJ, TP, OFC, and precuneus; however, unlike Bzdok et al. (2012), we did not detect significant amygdala involvement. This divergence may stem from differences in contrast selection—our analysis focused predominantly on moral versus control/neutral comparisons. Our conjunction results align with the findings reported by Sevinc and Spreng (2014), but we did not identify activation in the IFG or lingual gyrus. This discrepancy may be attributed to the larger number of contrasts included in our analysis (62 vs. 22 contrasts in their study), affording our study greater statistical power to detect consistent activation patterns (Müller et al. 2018).

Furthermore, our conjunction findings are consistent with those of Eres et al. (2018), who investigated morality using a broader set of search terms encompassing moral emotions (e.g., harm, guilt) and more diverse contrast types—such as medical versus non‐medical judgments, color versus emotion, and omission versus action. Importantly, our study adds nuance to this line of research by explicitly distinguishing between different types of moral tasks, thereby offering a more refined mapping of moral cognitive processes.

### 4.6 Limitations

Several limitations of this study should be acknowledged. First, due to the limited number of studies focusing on personal and impersonal moral dilemmas, we were unable to conduct separate analyses for these subtypes. Future research could explore the distinct neural mechanisms underlying these two kinds of moral processing. Second, while the dilemma moral tasks predominantly featured negative content valence (30 contrasts), the non‐dilemma tasks encompassed a broader range of valences (32 contrasts), including negative (12 contrasts), positive (13 contrasts), and mixed (7 contrasts) scenarios. Although we conducted ALE, conjunction, and contrast analyses for each content valence type within non‐dilemma tasks, the relatively small number of available contrasts for each valence category limited the robustness of these sub‐analyses. A similar concern applies to the perspective‑based sub‑analyses. In the dilemma tasks, the first‑person perspective was predominantly used (28/30 contrasts), whereas the third‑person perspective appeared only rarely (2/30 contrasts). Conversely, in the non‑dilemma tasks, the third‑person perspective was the most common (24/32 contrasts), while the first‑person perspective was used in only a minority of studies (8/32 contrasts). This notable asymmetry in perspective distribution across the two task types warrants caution when interpreting the present findings. Although we performed sub‑analyses for each perspective category, the comparisons (dilemma vs. non‑dilemma tasks within the first‑person perspective) did not yield significant results, likely due to the limited number of available studies in these cells. Future studies with larger datasets could clarify how scenario content valence and perspective category modulate neural responses in both types of moral tasks. Third, it is important to note that dilemma studies predominantly used non‑moral dilemma conditions as controls, whereas non‑dilemma studies largely employed neutral or semantic judgment tasks. This discrepancy in control conditions may affect the interpretability of our results, especially for non‑dilemma tasks. Future research would benefit from systematically examining the potential effects of different control conditions on moral judgment processing. Fourth, we acknowledge that databases such as PsycINFO, Scopus, and Embase represent important sources for meta‑analytic reviews. However, given that these databases are not openly accessible, the literature search in the present study was limited to publicly available databases. Future investigations should expand the search strategy to include these additional databases, thereby enhancing the comprehensiveness and generalizability of the findings. Fifth, similar to prior meta‐analyses (Garrigan et al. 2017), our study mainly included healthy adults, with limited representation of children and adolescents. Generalizing these findings to younger populations requires further empirical investigation. Sixth, socioeconomic status (SES) was rarely reported in the original studies, precluding analysis of its moderating role in moral decision‐making networks. Subsequent research could incorporate SES to better understand its influence on moral cognition. Finally, although we employed Neurosynth and BrainMap behavioral decoding to support functional interpretations, these methods rely on reverse inference. Direct empirical testing is needed to validate the involvement of specific behavioral and cognitive domains—such as social cognition, emotion, and value‐based decision‐making—in the identified neural networks.

## 5 Conclusions

The findings of the present meta‐analysis indicate that dilemma and non‐dilemma processing share a common neural architecture while also showing differential patterns of regional involvement. Specifically, non‐dilemma moral processing primarily recruits regions associated with affective valuation and rapid social appraisal, which support the computation of subjective moral value and access to autobiographical memory. In contrast, dilemma‐based tasks place heightened demands for conflict monitoring and mental state inference. Both types of tasks converge on a mesocorticolimbic network that subserves social cognition, value integration, emotional experience, and self‐referential processing. This overlap underscores the role of domain‐general mechanisms in moral cognition, while functional and connectivity differences reveal how task requirements shape the recruitment of complementary neural pathways. Taken together, these findings provide evidence consistent with the cognitive‐affective interaction model of moral decision‐making (Greene and Haidt 2002), suggesting that moral judgment may emerge from dynamic interactions among value‐based, affective, and mentalizing processes. The differential patterns observed between dilemma and non‐dilemma tasks further highlight the importance of considering moral context when investigating the neural basis of moral cognition. Future experimental and clinical research may benefit from explicitly distinguishing these task contexts when examining the cognitive and neural mechanisms underlying moral judgment.

## Author Contributions

**Junjun Fu**: methodology, formal analysis, writing – original draft. **Di Song**: formal analysis, writing – review and editing. **Serenella Tolomeo**: writing – review and editing. **Rongjun Yu**: conceptualization, supervision, writing – review and editing.

## Funding

Yu gratefully acknowledges the grants from the HKBU Research Committee (RC‐SFCRG/25‐26/R2/FASS/02), Hong Kong Research Grants Countil (N_HKBU218/23), and the Lee Shau Kee Foundation Limited for supporting this work.

## Conflicts of Interest

The authors declare no conflicts of interest.

## Supporting information




**Supporting File**: brb371864‐sup‐0001‐FigureS1.tif

**Supporting File**: brb371864‐sup‐0002‐FigureS2.tif

**Supporting File**: brb371864‐sup‐0003‐FigureS3.tif

**Supporting File**: brb371864‐sup‐0004‐FigureS4.tif

**Supporting File**: brb371864‐sup‐0005‐TableS1.xlsx

**Supporting File**: brb371864‐sup‐0006‐TableS2.xlsx

**Supporting File**: brb371864‐sup‐0007‐TableS3.xlsx

**Supporting File**: brb371864‐sup‐0008‐TableS4.xlsx

**Supporting File**: brb371864‐sup‐0009‐TableS5.xlsx

**Supporting File**: brb371864‐sup‐00010‐TableS6.xlsx

**Supporting File**: brb371864‐sup‐00011‐TableS7.xlsx

**Supporting File**: brb371864‐sup‐00012‐TableS8.xlsx

**Supporting File**: brb371864‐sup‐00013‐TableS9.xlsx

**Supporting File**: brb371864‐sup‐00014‐TableS10.xlsx

**Supporting File**: brb371864‐sup‐00015‐TableS11.xlsx

**Supporting File**: brb371864‐sup‐00016‐TableS12.xlsx

**Supporting File**: brb371864‐sup‐00017‐TableS13.xlsx

**Supporting File**: brb371864‐sup‐00018‐TableS14.docx

**Supporting File**: brb371864‐sup‐00019‐TableS15.docx

## Data Availability

This study is a meta‐analysis. All extracted data and code used in the current study are available at https://doi.org/10.17605/OSF.IO/X6AEW.

## References

[brb371864-bib-0001] Acar, F. , R. Seurinck , S. B. Eickhoff , and B. Moerkerke . 2018. “Assessing Robustness Against Potential Publication Bias in Activation Likelihood Estimation (ALE) Meta‐Analyses for fMRI.” PLoS ONE 13, no. 11: e0208177. 10.1371/journal.pone.0208177 PMC6267999 30500854

[brb371864-bib-0003] Amodio, D. M. , and C. D. Frith . 2006. “Meeting of Minds: The Medial Frontal Cortex and Social Cognition.” Nature Reviews Neuroscience 7, no. 4: 268–277. 10.1038/nrn1884. 16552413

[brb371864-bib-0004] Anderson, S. W. , A. Bechara , H. Damasio , D. Tranel , and A. R. Damasio . 1999. “Impairment of Social and Moral Behavior Related to Early Damage in Human Prefrontal Cortex.” Nature Neuroscience 2, no. 11: 1032–1037. 10.1038/14833. 10526345

[brb371864-bib-0005] Andrews‐Hanna, J. R. , J. Smallwood , and R. N. Spreng . 2014. “The Default Network and Self‐Generated Thought: Component Processes, Dynamic Control, and Clinical Relevance.” Annals of the New York Academy of Sciences 1316, no. 1: 29–52. 10.1111/nyas.12360. PMC4039623 24502540

[brb371864-bib-0006] Arioli, M. , M. P. M. Colletti , Z. Cattaneo , and N. Canessa . 2026. “Mapping the Functional Neurocognitive Correlates of Loss Aversion: A Coordinate‐Based Meta‐Analysis.” Neuroscience & Biobehavioral Reviews 00: 106937. 10.1016/j.neubiorev.2026.106937 42674197

[brb371864-bib-0007] Avram, M. , E. Gutyrchik , Y. Bao , E. Poeppel , M. Reiser , and J. Blautzik . 2013. “Neurofunctional Correlates of Esthetic and Moral Judgments.” Neuroscience Letters 534: 128–132. 10.1016/j.neulet.2012.11.053. 23262080

[brb371864-bib-0008] Avram, M. , K. Hennig‐Fast , Y. Bao , et al. 2014. “Neural Correlates of Moral Judgments in First‐and Third‐Person Perspectives: Implications for Neuroethics and Beyond.” Bmc Neuroscience 15, no. 1: 1–11. 10.1186/1471-2202-15-39. PMC3991864 24742205

[brb371864-bib-0009] Bahnemann, M. , I. Dziobek , K. Prehn , I. Wolf , and H. R. Heekeren . 2010. “Sociotopy in the Temporoparietal Cortex: Common Versus Distinct Processes.” Social Cognitive and Affective Neuroscience 5, no. 1: 48–58. 10.1093/scan/nsp045. PMC2840843 19966326

[brb371864-bib-0010] Ballesta, S. , W. Shi , K. E. Conen , and C. Padoa‐Schioppa . 2020. “Values Encoded in Orbitofrontal Cortex Are Causally Related to Economic Choices.” Nature 588, no. 7838: 450–453. 10.1038/s41586-020-2880-x. PMC7746614 33139951

[brb371864-bib-0011] Bartra, O. , J. T. McGuire , and J. W. Kable . 2013. “The Valuation System: A Coordinate‐Based Meta‐Analysis of BOLD fMRI Experiments Examining Neural Correlates of Subjective Value.” Neuroimage 76, no. 1: 412–427. 10.1016/j.neuroimage.2013.02.063. PMC3756836 23507394

[brb371864-bib-0012] Bellucci, G. , F. Molter , and S. Q. Park . 2019. “Neural Representations of Honesty Predict Future Trust Behavior.” Nature Communications 10, no. 1: 5184. 10.1038/s41467-019-13261-8. PMC6858375 31729396

[brb371864-bib-0013] Blair, J. , A. A. Marsh , E. Finger , K. S. Blair , and J. Luo . 2006. “Neuro‐Cognitive Systems Involved in Morality.” Philosophical Explorations 9, no. 1: 13–27. 10.1080/13869790500492359.

[brb371864-bib-0014] Borg, J. S. , C. Hynes , J. Van Horn , S. Grafton , and W. Sinnott‐Armstrong . 2006. “Consequences, Action, and Intention as Factors in Moral Judgments: An fMRI Investigation.” Journal of Cognitive Neuroscience 18, no. 5: 803–817. 10.1162/jocn.2006.18.5.803. 16768379

[brb371864-bib-0015] Buckholtz, J. W. , and R. Marois . 2012. “The Roots of Modern Justice: Cognitive and Neural Foundations of Social Norms and Their Enforcement.” Nature Neuroscience 15, no. 5: 655–661. 10.1038/nn.3087. 22534578

[brb371864-bib-0016] Buckner, R. L. , J. R. Andrews‐Hanna , and D. L. Schacter . 2008. “The Brain's Default Network—Anatomy, Function, and Relevance to Disease.” Annals of the New York Academy of Sciences 1124, no. 1: 1–38. 10.1196/annals.1440.011. 18400922

[brb371864-bib-0017] Bzdok, D. , L. Schilbach , K. Vogeley , et al. 2012. “Parsing the Neural Correlates of Moral Cognition: ALE Meta‐Analysis on Morality, Theory of Mind, and Empathy.” Brain Structure & Function 217, no. 4: 783–796. 10.1007/s00429-012-0380-y. PMC3445793 22270812

[brb371864-bib-0018] Camille, N. , G. Coricelli , J. Sallet , P. Pradat‐Diehl , J. R. Duhamel , and A. Sirigu . 2004. “The Involvement of the Orbitofrontal Cortex in the Experience of Regret.” Science 304, no. 5674: 1167–1170. 10.1126/science.1094550. 15155951

[brb371864-bib-0019] Carter, R. M. , and S. A. Huettel . 2013. “A Nexus Model of the Temporal‐Parietal Junction.” Trends in Cognitive Sciences 17, no. 7: 328–336. 10.1016/j.tics.2013.05.007. PMC3750983 23790322

[brb371864-bib-0020] Casebeer, W. D. 2003. “Moral Cognition and Its Neural Constituents.” Nature Reviews Neuroscience 4, no. 10: 840–846. 10.1038/nrn1223. 14523383

[brb371864-bib-0021] Casebeer, W. D. , and P. S. Churchland . 2003. “The Neural Mechanisms of Moral Cognition: A Multiple‐Aspect Approach to Moral Judgment and Decision‐Making.” Biology & Philosophy 18, no. 1: 169–194. 10.1023/a:1023380907603.

[brb371864-bib-0022] Cavanna, A. E. , and M. R. Trimble . 2006. “The Precuneus: A Review of Its Functional Anatomy and Behavioural Correlates.” Brain 129, no. Pt 3: 564–583. 10.1093/brain/awl004. 16399806

[brb371864-bib-0023] Cecchetto, C. , E. Lancini , D. Bueti , R. I. Rumiati , and V. Parma . 2019. “Body Odors (Even When Masked) Make You More Emotional: Behavioral and Neural Insights.” Scientific Reports 9: Article 5489. 10.1038/s41598-019-41937-0. PMC6445102 30940875

[brb371864-bib-0024] Chen, C. , R. M. Martinez , and Y. Cheng . 2020. “The Key to Group Fitness: The Presence of Another Synchronizes Moral Attitudes and Neural Responses During Moral Decision‐Making.” Neuroimage 213: 116732. 10.1016/j.neuroimage.2020.116732. 32173411

[brb371864-bib-0025] Cheng, Q. , X. Cui , J. Lin , X. Weng , and L. Mo . 2020. “Neural Correlates of Moral Goodness and Moral Beauty Judgments.” Brain Research 1726: 146534. 10.1016/j.brainres.2019.146534. 31669285

[brb371864-bib-0026] Chiong, W. , S. M. Wilson , M. D'Esposito , et al. 2013. “The Salience Network Causally Influences Default Mode Network Activity During Moral Reasoning.” Brain 136: 1929–1941. 10.1093/brain/awt066. PMC3673466 23576128

[brb371864-bib-0027] Cui, F. , X. Huang , X. Li , C. Liao , J. Liu , and Y. J. Luo . 2021. “Moral Conflict in Economic Decision Making: The Role of the Anterior Cingulate Cortex‐Striatum Pathway.” Cerebral Cortex 31, no. 11: 5121–5130. 10.1093/cercor/bhab146. 34148081

[brb371864-bib-0028] Cui, W. , J. Zhu , Y. Long , et al. 2026. “Robust Lesion Network Mapping Reveals Genuine Symptom‐Specific Networks.” BioRxiv 00: 00.

[brb371864-bib-0029] Cushman, F. 2008. “Crime and Punishment: Distinguishing the Roles of Causal and Intentional Analyses in Moral Judgment.” Cognition 108, no. 2: 353–380. 10.1016/j.cognition.2008.03.006. 18439575

[brb371864-bib-0030] Damasio, A. R. 1996. “The Somatic Marker Hypothesis and the Possible Functions of the Prefrontal Cortex.” Philosophical Transactions of the Royal Society B‐Biological Sciences 351, no. 1346: 1413–1420. 10.1098/rstb.1996.0125. 8941953

[brb371864-bib-0031] de Achával, D. , M. F. Villarreal , A. Salles , et al. 2013. “Activation of Brain Areas Concerned With Social Cognition During Moral Decisions Is Abnormal in Schizophrenia Patients and Unaffected Siblings.” Journal of Psychiatric Research 47, no. 6: 774–782. 10.1016/j.jpsychires.2012.12.018. 23403281

[brb371864-bib-0032] den Ouden, H. E. M. , U. Frith , C. Frith , and S. J. Blakemore . 2005. “Thinking About Intentions.” Neuroimage 28, no. 4: 787–796. 10.1016/j.neuroimage.2005.05.001. 15964210

[brb371864-bib-0033] Döhnel, K. , T. Schuwerk , J. Meinhardt , B. Sodian , G. Hajak , and M. Sommer . 2012. “Functional Activity of the Right Temporo‐Parietal Junction and of the Medial Prefrontal Cortex Associated With True and False Belief Reasoning.” Neuroimage 60, no. 3: 1652–1661. 10.1016/j.neuroimage.2012.01.073. 22300812

[brb371864-bib-0034] Eickhoff, S. B. , D. Bzdok , A. R. Laird , et al. 2011. “Co‐Activation Patterns Distinguish Cortical Modules, Their Connectivity and Functional Differentiation.” Neuroimage 57, no. 3: 938–949. 10.1016/j.neuroimage.2011.05.021. PMC3129435 21609770

[brb371864-bib-0035] Eickhoff, S. B. , D. Bzdok , A. R. Laird , F. Kurth , and P. T. Fox . 2012. “Activation Likelihood Estimation Meta‐Analysis Revisited.” Neuroimage 59, no. 3: 2349–2361. 10.1016/j.neuroimage.2011.09.017. PMC3254820 21963913

[brb371864-bib-0036] Eickhoff, S. B. , A. R. Laird , C. Grefkes , L. E. Wang , K. Zilles , and P. T. Fox . 2009. “Coordinate‐Based Activation Likelihood Estimation Meta‐Analysis of Neuroimaging Data: A Random‐Effects Approach Based on Empirical Estimates of Spatial Uncertainty.” Human Brain Mapping 30, no. 9: 2907–2926. 10.1002/hbm.20718. PMC2872071 19172646

[brb371864-bib-0037] Eickhoff, S. B. , T. E. Nichols , A. R. Laird , et al. 2016. “Behavior, Sensitivity, and Power of Activation Likelihood Estimation Characterized by Massive Empirical Simulation.” Neuroimage 137: 70–85. 10.1016/j.neuroimage.2016.04.072. PMC4981641 27179606

[brb371864-bib-0038] Ellis, A. W. , A. W. Young , and E. M. Critchley . 1989. “Loss of Memory for People Following Temporal Lobe Damage.” Brain 112, no. Pt 6: 1469–1483. 10.1093/brain/112.6.1469. 2597991

[brb371864-bib-0039] Eres, R. , W. R. Louis , and P. Molenberghs . 2018. “Common and Distinct Neural Networks Involved in fMRI Studies Investigating Morality: An ALE Meta‐Analysis.” Social Neuroscience 13, no. 4: 384–398. 10.1080/17470919.2017.1357657. 28724332

[brb371864-bib-0040] Fede, S. J. , and K. A. Kiehl . 2020. “Meta‐Analysis of the Moral Brain: Patterns of Neural Engagement Assessed Using Multilevel Kernel Density Analysis.” Brain Imaging and Behavior 14, no. 2: 534–547. 10.1007/s11682-019-00035-5. 30706370

[brb371864-bib-0041] FeldmanHall, O. , T. Dalgleish , R. Thompson , D. Evans , S. Schweizer , and D. Mobbs . 2012. “Differential Neural Circuitry and Self‐Interest in Real vs Hypothetical Moral Decisions.” Social Cognitive and Affective Neuroscience 7, no. 7: 743–751. 10.1093/scan/nss069. PMC3475363 22711879

[brb371864-bib-0042] FeldmanHall, O. , D. Mobbs , and T. Dalgleish . 2014. “Deconstructing the Brain's Moral Network: Dissociable Functionality Between the Temporoparietal Junction and Ventro‐Medial Prefrontal Cortex.” Social Cognitive and Affective Neuroscience 9, no. 3: 297–306. 10.1093/scan/nss139. PMC3980797 23322890

[brb371864-bib-0043] Fink, G. R. , H. J. Markowitsch , M. Reinkemeier , T. Bruckbauer , J. Kessler , and W. D. Heiss . 1996. “Cerebral Representation of One's Own Past: Neural Networks Involved in Autobiographical Memory.” Journal of Neuroscience 16, no. 13: 4275–4282. 10.1523/jneurosci.16-13-04275.1996. PMC6579004 8753888

[brb371864-bib-0044] Fox, M. D. , A. Z. Snyder , J. L. Vincent , M. Corbetta , D. C. Van Essen , and M. E. Raichle . 2005. “The Human Brain Is Intrinsically Organized Into Dynamic, Anticorrelated Functional Networks.” Proceedings of the National Academy of Sciences of the United States of America 102, no. 27: 9673–9678. 10.1073/pnas.0504136102. PMC1157105 15976020

[brb371864-bib-0045] Freton, M. , C. Lemogne , L. Bergouignan , P. Delaveau , S. Lehericy , and P. Fossati . 2014. “The Eye of the Self: Precuneus Volume and Visual Perspective During Autobiographical Memory Retrieval.” Brain Structure & Function 219, no. 3: 959–968. 10.1007/s00429-013-0546-2. 23553546

[brb371864-bib-0046] Frith, U. , and C. D. Frith . 2003. “Development and Neurophysiology of Mentalizing.” Philosophical Transactions of the Royal Society B‐Biological Sciences 358, no. 1431: 459–473. 10.1098/rstb.2002.1218. PMC1693139 12689373

[brb371864-bib-0047] Fumagalli, M. , and A. Priori . 2012. “Functional and Clinical Neuroanatomy of Morality.” Brain 135: 2006–2021. 10.1093/brain/awr334. 22334584

[brb371864-bib-0048] Garrigan, B. , A. L. R. Adlam , and P. E. Langdon . 2017. “The Neural Correlates of Moral Decision‐Making: a Systematic Review and Meta‐Analysis of Moral Evaluations and Response Decision Judgements (vol 108, pg 88, 2016).” Brain and Cognition 111: 104–106. 10.1016/j.bandc.2016.10.002. 27566002

[brb371864-bib-0049] Goodenough, O. R. , and K. Prehn . 2004. “A Neuroscientific Approach to Normative Judgment in Law and Justice.” Philosophical Transactions of the Royal Society B – Biological Sciences 359, no. 1451: 1709–1726. 10.1098/rstb.2004.1552. PMC1693459 15590612

[brb371864-bib-0050] Gorno‐Tempini, M. L. , C. J. Price , O. Josephs , et al. 1998. “The Neural Systems Sustaining Face and Proper‐Name Processing.” Brain 121, no. 11: 2103–2118. 10.1093/brain/121.11.2103. 9827770

[brb371864-bib-0051] Greene, J. , and J. Haidt . 2002. “How (and Where) Does Moral Judgment Work?” Trends in Cognitive Sciences 6, no. 12: 517–523. 10.1016/S1364-6613(02)0201-9. 12475712

[brb371864-bib-0052] Greene, J. D. , L. E. Nystrom , A. D. Engell , J. M. Darley , and J. D. Cohen . 2004. “The Neural Bases of Cognitive Conflict and Control in Moral Judgment.” Neuron 44, no. 2: 389–400. 10.1016/j.neuron.2004.09.027. 15473975

[brb371864-bib-0053] Greene, J. D. , R. B. Sommerville , L. E. Nystrom , J. M. Darley , and J. D. Cohen . 2001. “An fMRI Investigation of Emotional Engagement in Moral Judgment.” Science 293, no. 5537: 2105–2108. 10.1126/science.1062872. 11557895

[brb371864-bib-0054] Gu, R. , W. Huang , J. Camilleri , et al. 2019. “Love Is Analogous to Money in Human Brain: Coordinate‐Based and Functional Connectivity Meta‐Analyses of Social and Monetary Reward Anticipation.” Neuroscience and Biobehavioral Reviews 100: 108–128. 10.1016/j.neubiorev.2019.02.017. PMC7250476 30807783

[brb371864-bib-0055] Haidt, J. 2001. “The Emotional Dog and Its Rational Tail: A Social Intuitionist Approach to Moral Judgment.” Psychological Review 108, no. 4: 814–834. 10.1037//0033-295x.108.4.814. 11699120

[brb371864-bib-0056] Hampton, A. N. , P. Bossaerts , and J. P. O'Doherty . 2008. “Neural Correlates of Mentalizing‐Related Computations During Strategic Interactions in Humans.” Proceedings of the National Academy of Sciences of the United States of America 105, no. 18: 6741–6746. 10.1073/pnas.0711099105. PMC2373314 18427116

[brb371864-bib-0057] Han, H. , J. Chen , C. Jeong , and G. H. Glover . 2016. “Influence of the Cortical Midline Structures on Moral Emotion and Motivation in Moral Decision‐Making.” Behavioural Brain Research 302: 237–251. 10.1016/j.bbr.2016.01.001. 26772629

[brb371864-bib-0058] Han, H. , G. H. Glover , and C. Jeong . 2014. “Cultural Influences on the Neural Correlate of Moral Decision‐Making Processes.” Behavioural Brain Research 259: 215–228. 10.1016/j.bbr.2013.11.012. 24263193

[brb371864-bib-0059] Harada, T. , S. Itakura , F. Xu , et al. 2009. “Neural Correlates of the Judgment of Lying: A Functional Magnetic Resonance Imaging Study.” Neuroscience Research 63, no. 1: 24–34. 10.1016/j.neures.2008.09.010. 18992288

[brb371864-bib-0060] Harenski, C. L. , O. Antonenko , M. S. Shane , and K. A. Kiehl . 2008. “Gender Differences in Neural Mechanisms Underlying Moral Sensitivity.” Social Cognitive and Affective Neuroscience 3, no. 4: 313–321. 10.1093/scan/nsn026. PMC2607058 19015084

[brb371864-bib-0061] Harenski, C. L. , K. A. Harenski , M. S. Shane , and K. A. Kiehl . 2012. “Neural Development of Mentalizing in Moral Judgment From Adolescence to Adulthood.” Developmental Cognitive Neuroscience 2, no. 1: 162–173. 10.1016/j.dcn.2011.09.002. PMC3259704 22267967

[brb371864-bib-0062] Harrison, B. J. , J. Pujol , C. Soriano‐Mas , et al. 2012. “Neural Correlates of Moral Sensitivity in Obsessive‐Compulsive Disorder.” Archives of General Psychiatry 69, no. 7: 741–749. 10.1001/archgenpsychiatry.2011.2165. 22752238

[brb371864-bib-0063] Heekeren, H. R. , I. Wartenburger , H. Schmidt , K. Prehn , H. P. Schwintowski , and A. Villringer . 2005. “Influence of Bodily Harm on Neural Correlates of Semantic and Moral Decision‐Making.” Neuroimage 24, no. 3: 887–897. 10.1016/j.neuroimage.2004.09.026. 15652323

[brb371864-bib-0064] Heekeren, H. R. , I. Wartenburger , H. Schmidt , H. P. Schwintowski , and A. Villringer . 2003. “An fMRI Study of Simple Ethical Decision‐Making.” Neuroreport 14, no. 9: 1215–1219. 10.1097/00001756-200307010-00005. 12824762

[brb371864-bib-0066] Hill, C. A. , S. Suzuki , R. Polania , M. Moisa , J. P. O'doherty , and C. C. Ruff . 2017. “A Causal Account of the Brain Network Computations Underlying Strategic Social Behavior.” Nature Neuroscience 20, no. 8: 1142–1149. 10.1038/nn.4602. 28692061

[brb371864-bib-0067] Holmes, C. J. , R. Hoge , L. Collins , and A. C. Evans . 1996. “Enhancement of T1 MR Images Using Registration for Signal Averaging.” Neuroimage 3, no. 3: S28. 10.1016/S1053-8119(96)80030-9. 9530404

[brb371864-bib-0068] Hu, Y. , A. M. Pereira , X. Gao , et al. 2021. “Right Temporoparietal Junction Underlies Avoidance of Moral Transgression in Autism Spectrum Disorder.” Journal of Neuroscience 41, no. 8: 1699–1715. 10.1523/JNEUROSCI.1237-20.2020. PMC8115877 33158960

[brb371864-bib-0069] Hume, D. 1969/1777. An Enquiry Concerning the Principles of Morals. Open Court. (Original work published 1777).

[brb371864-bib-0070] Kant, I. 1785/1993. Grounding for the Metaphysics of Morals. Hackett Publishing Co.

[brb371864-bib-0071] Killen, M. , K. L. Mulvey , C. Richardson , N. Jampol , and A. Woodward . 2011. “The Accidental Transgressor: Morally‐Relevant Theory of Mind.” Cognition 119, no. 2: 197–215. 10.1016/j.cognition.2011.01.006. PMC4142201 21377148

[brb371864-bib-0072] Kircher, T. T. J. , C. Senior , M. L. Phillips , et al. 2000. “Towards a Functional Neuroanatomy of Self Processing: Effects of Faces and Words.” Cognitive Brain Research 10, no. 1‐2: 133–144. 10.1016/s0926-6410(00)00036-7. 10978701

[brb371864-bib-0073] Knobe, J. 2005. “Theory of Mind and Moral Cognition: Exploring the Connections.” Trends in Cognitive Sciences 9, no. 8: 357–359. 10.1016/j.tics.2005.06.011. 16006176

[brb371864-bib-0074] Koenigs, M. , and D. Tranel . 2007. “Irrational Economic Decision‐Making After Ventromedial Prefrontal Damage: Evidence From the Ultimatum Game.” Journal of Neuroscience 27, no. 4: 951–956. 10.1523/jneurosci.4606-06.2007. PMC2490711 17251437

[brb371864-bib-0075] Koenigs, M. , L. Young , R. Adolphs , et al. 2007. “Damage to the Prefrontal Cortex Increases Utilitarian Moral Judgements.” Nature 446, no. 7138: 908–911. 10.1038/nature05631. PMC2244801 17377536

[brb371864-bib-0076] Kohlberg, L. 1963. “Moral Development and Identification.” In Child Psychology: The Sixty‐Second Yearbook of the National Society for the Study of Education, Part 1, edited by H. W. Stevenson , J. Kagan , C. Spiker , N. B. Henry , and H. G. Richey , 277–332. National Society for the Study of Education; The University of Chicago Press. 10.1037/13101-008.

[brb371864-bib-0077] Koster‐Hale, J. , R. Saxe , J. Dungan , and L. L. Young . 2013. “Decoding Moral Judgments From Neural Representations of Intentions.” Proceedings of the National Academy of Sciences of the United States of America 110, no. 14: 5648–5653. 10.1073/pnas.1207992110. PMC3619352 23479657

[brb371864-bib-0078] Laird, A. R. , S. B. Eickhoff , F. Kurth , et al. 2009. “ALE Meta‐Analysis Workflows via the Brainmap Database: Progress Towards a Probabilistic Functional Brain Atlas.” Frontiers in neuroinformatics 3: 598. 10.3389/neuro.11.023.2009. PMC2715269 19636392

[brb371864-bib-0079] Laird, A. R. , P. M. Fox , C. J. Price , et al. 2005. “ALE Meta‐Analysis: Controlling the False Discovery Rate and Performing Statistical Contrasts.” Human Brain Mapping 25, no. 1: 155–164. 10.1002/hbm.20136. PMC6871747 15846811

[brb371864-bib-0080] Lancaster, J. L. , A. R. Laird , S. B. Eickhoff , M. J. Martinez , P. M. Fox , and P. T. Fox . 2012. “Automated Regional Behavioral Analysis for Human Brain Images.” Frontiers in Neuroinformatics 6: 23. 10.3389/fninf.2012.00023. PMC3428588 22973224

[brb371864-bib-0081] Lancaster, J. L. , D. Tordesillas‐Gutierrez , M. Martinez , et al. 2007. “Bias Between MNI and Talairach Coordinates Analyzed Using the ICBM‐152 Brain Template.” Human Brain Mapping 28, no. 11: 1194–1205. 10.1002/hbm.20345. PMC6871323 17266101

[brb371864-bib-0082] Langner, R. , S. Leiberg , F. Hoffstaedter , and S. B. Eickhoff . 2018. “Towards a Human Self‐Regulation System: Common and Distinct Neural Signatures of Emotional and Behavioural Control.” Neuroscience and Biobehavioral Reviews 90: 400–410. 10.1016/j.neubiorev.2018.04.022. PMC5994341 29730485

[brb371864-bib-0083] Langner, R. , C. Rottschy , A. R. Laird , P. T. Fox , and S. B. Eickhoff . 2014. “Meta‐Analytic Connectivity Modeling Revisited: Controlling for Activation Base Rates.” Neuroimage 99: 559–570. 10.1016/j.neuroimage.2014.06.007. PMC4112007 24945668

[brb371864-bib-0084] Li, X. , J. Yang , P. Li , and H. Li . 2016. “Individual Differences in Moral Judgment Competence Are Related to Activity of the Prefrontal Cortex When Attributing Blame to Evil Intention.” Social Neuroscience 11, no. 4: 438–448. 10.1080/17470919.2015.1093960. 26569419

[brb371864-bib-0085] Lieberman, M. D. 2007. “Social Cognitive Neuroscience: A Review of Core Processes.” Annual Review of Psychology 58: 259–289. 10.1146/annurev.psych.58.110405.085654. 17002553

[brb371864-bib-0086] Lim, J. , Y. A. Kurnianingsih , H. H. Ong , and O. D. A. Mullette‐Gillman . 2017. “Moral Judgment Modulation by Disgust Priming via Altered Fronto‐Temporal Functional Connectivity.” Scientific Reports 7: Article 10887. 10.1038/s41598-017-11147-7. PMC5589926 28883626

[brb371864-bib-0087] Liu, J. , C. Liao , J. Lu , Y.‐j. Luo , and F. Cui . 2019. “Moral Contagion: Devaluation Effect of Immorality on Hypothetical Judgments of Economic Value.” Human Brain Mapping 40, no. 7: 2076–2088. 10.1002/hbm.24508. PMC6865687 30624839

[brb371864-bib-0088] Luo, Q. , M. Nakic , T. Wheatley , R. Richell , A. Martin , and R. J. R. Blair . 2006. “The Neural Basis of Implicit Moral Attitude—An IAT Study Using Event‐Related fMRI.” Neuroimage 30, no. 4: 1449–1457. 10.1016/j.neuroimage.2005.11.005. 16418007

[brb371864-bib-0089] Marazziti, D. , S. Baroni , P. Landi , D. Ceresoli , and L. Dell'Osso . 2013. “The Neurobiology of Moral Sense: Facts or Hypotheses?” Annals of General Psychiatry 12: Article 6. 10.1186/1744-859x-12-6. PMC3616987 23497376

[brb371864-bib-0090] Markowitsch, H. J. 1995. “Which Brain Regions Are Critically Involved in the Retrieval of Old Episodic Memory?” Brain Research Brain Research Reviews 21, no. 2: 117–127. 10.1016/0165-0173(95)00007-0. 8866670

[brb371864-bib-0091] Markowitsch, H. J. , P. Calabrese , J. Liess , M. Haupts , H. F. Durwen , and W. Gehlen . 1993. “Retrograde Amnesia After Traumatic Injury of the Fronto‐Temporal Cortex.” Journal of Neurology, Neurosurgery, and Psychiatry 56, no. 9: 988–992. 10.1136/jnnp.56.9.988. PMC489734 8410040

[brb371864-bib-0092] Moher, D. , A. Liberati , J. Tetzlaff , D. G. Altman , and P. Grp . 2009. “Preferred Reporting Items for Systematic Reviews and Meta‐Analyses: the PRISMA Statement.” Bmj‐British Medical Journal 339: b2535. 10.1136/bmj.b2535. PMC2714657 19622551

[brb371864-bib-0093] Moll, J. , R. de Oliveira‐Souza , I. E. Bramati , and J. Grafman . 2002a. “Functional Networks in Emotional Moral and Nonmoral Social Judgments.” Neuroimage 16, no. 3: 696–703. 10.1006/nimg.2002.1118. 12169253

[brb371864-bib-0094] Moll, J. , R. de Oliveira‐Souza , P. J. Eslinger , et al. 2002b. “The Neural Correlates of Moral Sensitivity: A Functional Magnetic Resonance Imaging Investigation of Basic and Moral Emotions.” Journal of Neuroscience 22, no. 7: 2730–2736. 10.1523/jneurosci.22-07-02730.2002. PMC6758288 11923438

[brb371864-bib-0095] Moll, J. , R. de Oliveira‐Souza , and P. J. Eslinger . 2003. “Morals and the Human Brain: A Working Model.” Neuroreport 14, no. 3: 299–305. 10.1097/01.wnr.0000057866.05120.28. 12634472

[brb371864-bib-0096] Moll, J. , R. de Oliveira‐Souza , F. T. Moll , et al. 2005. “The Moral Affiliations of Disgust—A Functional MRI Study.” Cognitive and Behavioral Neurology 18, no. 1: 68–78. 10.1097/01.wnn.0000152236.46475.a7. 15761278

[brb371864-bib-0097] Moll, J. , P. J. Eslinger , and R. D. Oliveira‐Souza . 2001. “Frontopolar and Anterior Temporal Cortex Activation in a Moral Judgment Task: Preliminary Functional MRI Results in Normal Subjects.” Arquivos De Neuro‐Psiquiatria 59, no. 3B: 654–664. 10.1590/S0004-282x2001000500001. 11593260

[brb371864-bib-0098] Monin, B. , D. A. Pizarro , and J. S. Beer . 2007. “Deciding Versus Reacting: Conceptions of Moral Judgment and the Reason‐Affect Debate.” Review of General Psychology 11, no. 2: 99–111. 10.1037/1089-2680.11.2.99.

[brb371864-bib-0099] Müller, V. I. , E. C. Cieslik , A. R. Laird , et al. 2018. “Ten Simple Rules for Neuroimaging Meta‐Analysis.” Neuroscience and Biobehavioral Reviews 84: 151–161. 10.1016/j.neubiorev.2017.11.012. PMC5918306 29180258

[brb371864-bib-0100] Nakamura, K. , R. Kawashima , N. Sato , et al. 2000. “Functional Delineation of the Human Occipito‐Temporal Areas Related to Face and Scene Processing. A PET Study.” Brain 123, no. Pt 9: 1903–1912. 10.1093/brain/123.9.1903. 10960054

[brb371864-bib-0101] Nakamura, K. , and K. Kubota . 1996. “The Primate Temporal Pole: Its Putative Role in Object Recognition and Memory.” Behavioural Brain Research 77, no. 1‐2: 53–77. 10.1016/0166-4328(95)00227-8. 8762159

[brb371864-bib-0102] Ngo, T. , M. Ghio , L. Kuchinke , P. Roser , and C. Bellebaum . 2019. “Moral Decision Making Under modafinil: A Randomized Placebo‐Controlled Double‐Blind Crossover fMRI Study.” Psychopharmacology 236, no. 9: 2747–2759. 10.1007/s00213-019-05250-y. 31037409

[brb371864-bib-0103] Nichols, T. , M. Brett , J. Andersson , T. Wager , and J. B. Poline . 2005. “Valid Conjunction Inference With the Minimum Statistic.” Neuroimage 25, no. 3: 653–660. 10.1016/j.neuroimage.2004.12.005. 15808966

[brb371864-bib-0104] Obeso, I. , M. Moisa , C. C. Ruff , and J.‐C. Dreher . 2018. “A Causal Role for Right Temporo‐Parietal Junction in Signaling Moral Conflict.” Elife 7: e40671. 10.7554/eLife.40671. PMC6298767 30561334

[brb371864-bib-0105] Padoa‐Schioppa, C. , and J. A. Assad . 2006. “Neurons in the Orbitofrontal Cortex Encode Economic Value.” Nature 441, no. 7090: 223–226. 10.1038/nature04676. PMC2630027 16633341

[brb371864-bib-0106] Page, M. J. , J. E. McKenzie , P. M. Bossuyt , et al. 2021. “The PRISMA 2020 Statement: An Updated Guideline for Reporting Systematic Reviews.” International Journal of Surgery 88: 105906. 10.1016/j.ijsu.2021.105906 33789826

[brb371864-bib-0107] Parkinson, C. , W. Sinnott‐Armstrong , P. E. Koralus , A. Mendelovici , V. McGeer , and T. Wheatley . 2011. “Is Morality Unified? Evidence That Distinct Neural Systems Underlie Moral Judgments of Harm, Dishonesty, and Disgust.” Journal of cognitive neuroscience 23, no. 10: 3162–3180. 10.1162/jocn_a_00017. 21452951

[brb371864-bib-0108] Peng, S. , P. Xu , Y. Jiang , and G. Gong . 2022. “Activation Network Mapping for Integration of Heterogeneous fMRI Findings.” Nature Human Behaviour 6, no. 10: 1417–1429. 10.1038/s41562-022-01371-1. 35654963

[brb371864-bib-0109] Poldrack, R. 2006. “Can Cognitive Processes be Inferred From Neuroimaging Data?” Trends in Cognitive Sciences 10, no. 2: 59–63. 10.1016/j.tics.2005.12.004. 16406760

[brb371864-bib-0110] Preckel, K. , D. Scheele , M. Eckstein , W. Maier , and R. Hurlemann . 2015. “The Influence of Oxytocin on Volitional and Emotional Ambivalence.” Social Cognitive and Affective Neuroscience 10, no. 7: 987–993. 10.1093/scan/nsu147. PMC4483569 25398434

[brb371864-bib-0111] Prehn, K. , I. Wartenburger , K. Meriau , et al. 2008. “Individual Differences in Moral Judgment Competence Influence Neural Correlates of Socio‐Normative Judgments.” Social Cognitive and Affective Neuroscience 3, no. 1: 33–46. 10.1093/scan/nsm037. PMC2569820 19015093

[brb371864-bib-0112] Pujol, J. , I. Batalla , O. Contreras‐Rodriguez , et al. 2012. “Breakdown in the Brain Network Subserving Moral Judgment in Criminal Psychopathy.” Social Cognitive and Affective Neuroscience 7, no. 8: 917–923. 10.1093/scan/nsr075. PMC3501707 22037688

[brb371864-bib-0113] Qu, C. , J. Bénistant , and J.‐C. Dreher . 2022. “Neurocomputational Mechanisms Engaged in Moral Choices and Moral Learning.” Neuroscience and Biobehavioral Reviews 132: 50–60. 10.1016/j.neubiorev.2021.11.023. 34826508

[brb371864-bib-0114] Qu, C. , Y. Hu , Z. Tang , E. Derrington , and J.‐C. Dreher . 2020. “Neurocomputational Mechanisms Underlying Immoral Decisions Benefiting Self or Others.” Social Cognitive and Affective Neuroscience 15, no. 2: 135–149. 10.1093/scan/nsaa029. PMC7304519 32163158

[brb371864-bib-0115] Qu, C. , E. Météreau , L. Butera , M. C. Villeval , and J.‐C. Dreher . 2019. “Neurocomputational Mechanisms at Play When Weighing Concerns for Extrinsic Rewards, Moral Values, and Social Image.” PLoS Biology 17, no. 6: e3000283. 10.1371/journal.pbio.3000283. PMC6553686 31170138

[brb371864-bib-0116] Raichle, M. E. , A. M. MacLeod , A. Z. Snyder , W. J. Powers , D. A. Gusnard , and G. L. Shulman . 2001. “A Default Mode of Brain Function.” Proceedings of the National Academy of Sciences of the United States of America 98, no. 2: 676–682. 10.1073/pnas.98.2.676. PMC14647 11209064

[brb371864-bib-0117] Raine, A. , and Y. Yang . 2006. “Neural Foundations to Moral Reasoning and Antisocial Behavior.” Social Cognitive and Affective Neuroscience 1, no. 3: 203–213. 10.1093/scan/nsl033. PMC2555414 18985107

[brb371864-bib-0118] Reniers, R. L. E. P. , R. Corcoran , B. A. Voellm , A. Mashru , R. Howard , and P. F. Liddle . 2012. “Moral Decision‐Making, ToM, Empathy and the Default Mode Network.” Biological Psychology 90, no. 3: 202–210. 10.1016/j.biopsycho.2012.03.009. 22459338

[brb371864-bib-0119] Rich, E. L. , and J. D. Wallis . 2016. “Decoding Subjective Decisions From Orbitofrontal Cortex.” Nature Neuroscience 19, no. 7: 973–980. 10.1038/nn.4320. PMC4925198 27273768

[brb371864-bib-0120] Robertson, D. , J. Snarey , O. Ousley , et al. 2007. “The Neural Processing of Moral Sensitivity to Issues of Justice and Care.” Neuropsychologia 45, no. 4: 755–766. 10.1016/j.neuropsychologia.2006.08.014. 17174987

[brb371864-bib-0121] Rudebeck, P. H. , and E. A. Murray . 2014. “The Orbitofrontal Oracle: Cortical Mechanisms for the Prediction and Evaluation of Specific Behavioral Outcomes.” Neuron 84, no. 6: 1143–1156. 10.1016/j.neuron.2014.10.049. PMC4271193 25521376

[brb371864-bib-0122] Ruff, C. C. , and E. Fehr . 2014. “The Neurobiology of Rewards and Values in Social Decision Making.” Nature Reviews Neuroscience 15, no. 8: 549–562. 10.1038/nrn3776. 24986556

[brb371864-bib-0123] Samartsidis, P. , C. R. Eickhoff , S. B. Eickhoff , et al. 2019. “Bayesian Log‐Gaussian Cox Process Regression: Applications to Meta‐Analysis of Neuroimaging Working Memory Studies.” Journal of the Royal Statistical Society Series C: Applied Statistics 68, no. 1: 217–234. 10.1111/rssc.12295 PMC6430202 30906075

[brb371864-bib-0124] Saver, J. L. , and A. R. Damasio . 1991. “Preserved Access and Processing of Social Knowledge in a Patient With Acquired Sociopathy due to Ventromedial Frontal Damage.” Neuropsychologia 29, no. 12: 1241–1249. 10.1016/0028-3932(91)90037-9. 1791934

[brb371864-bib-0125] Saxe, R. , D. K. Xiao , G. Kovacs , D. I. Perrett , and N. Kanwisher . 2004. “A Region of Right Posterior Superior Temporal Sulcus Responds to Observed Intentional Actions.” Neuropsychologia 42, no. 11: 1435–1446. 10.1016/j.neuropsychologia.2004.04.015. 15246282

[brb371864-bib-0126] Schaich Borg, J. , C. Hynes , J. Van Horn , S. Grafton , and W. Sinnott‐Armstrong . 2006. “Consequences, Action, and Intention as Factors in Moral Judgments: An fMRI Investigation.” Journal of Cognitive Neuroscience 18, no. 5: 803–817. 10.1162/jocn.2006.18.5.803. 16768379

[brb371864-bib-0127] Schaich Borg, J. , D. Lieberman , and K. A. Kiehl . 2008. “Infection, Incest, and Iniquity: Investigating the Neural Correlates of Disgust and Morality.” Journal of Cognitive Neuroscience 20, no. 9: 1529–1546. 10.1162/jocn.2008.20109. PMC3969035 18345982

[brb371864-bib-0128] Schleim, S. , T. M. Spranger , S. Erk , and H. Walter . 2011. “From Moral to Legal Judgment: The Influence of Normative Context in Lawyers and Other Academics.” Social Cognitive and Affective Neuroscience 6, no. 1: 48–57. 10.1093/scan/nsq010. PMC3023080 20194515

[brb371864-bib-0129] Schneider, K. , K. D. Pauly , A. Gossen , et al. 2013. “Neural Correlates of Moral Reasoning in Autism Spectrum Disorder.” Social Cognitive and Affective Neuroscience 8, no. 6: 702–710. 10.1093/scan/nss051. PMC3739915 22569187

[brb371864-bib-0130] Schuwerk, T. , K. Doehnel , B. Sodian , I. R. Keck , R. Rupprecht , and M. Sommer . 2014. “Functional Activity and Effective Connectivity of the Posterior Medial Prefrontal Cortex During Processing of Incongruent Mental States.” Human Brain Mapping 35, no. 7: 2950–2965. 10.1002/hbm.22377. PMC6869201 24115202

[brb371864-bib-0131] Sevinc, G. , H. Gurvit , and R. N. Spreng . 2017. “Salience Network Engagement With the Detection of Morally Laden Information.” Social Cognitive and Affective Neuroscience 12, no. 7: 1118–1127. 10.1093/scan/nsx035. PMC5490682 28338944

[brb371864-bib-0132] Sevinc, G. , and R. N. Spreng . 2014. “Contextual and Perceptual Brain Processes Underlying Moral Cognition: A Quantitative Meta‐Analysis of Moral Reasoning and Moral Emotions.” PLoS ONE 9, no. 2: Article e87427. 10.1371/journal.pone.0087427. PMC3913597 24503959

[brb371864-bib-0133] Sharvit, G. , E. Lin , P. Vuilleumier , and C. Corradi‐Dell'Acqua . 2020. “Does Inappropriate Behavior Hurt or Stink? The Interplay Between Neural Representations of Somatic Experiences and Moral Decisions.” Science Advances 6, no. 42: eaat4390. 10.1126/sciadv.aat4390. PMC7567598 33067240

[brb371864-bib-0134] Shenhav, A. , M. M. Botvinick , and J. D. Cohen . 2013. “The Expected Value of Control: An Integrative Theory of Anterior Cingulate Cortex Function.” Neuron 79, no. 2: 217–240. 10.1016/j.neuron.2013.07.007. PMC3767969 23889930

[brb371864-bib-0135] Shenhav, A. , and J. D. Greene . 2014. “Integrative Moral Judgment: Dissociating the Roles of the Amygdala and Ventromedial Prefrontal Cortex.” Journal of Neuroscience 34, no. 13: 4741–4749. 10.1523/jneurosci.3390-13.2014. PMC6608126 24672018

[brb371864-bib-0136] Smith, L. J. , P. Anand , A. Benattayallah , and T. L. Hodgson . 2015. “An fMRI Investigation of Moral Cognition in Healthcare Decision Making.” Journal of Neuroscience Psychology and Economics 8, no. 2: 116–133. 10.1037/npe0000038.

[brb371864-bib-0137] Sommer, M. , J. Meinhardt , C. Rothmayr , et al. 2014. “Me or You? Neural Correlates of Moral Reasoning in Everyday Conflict Situations in Adolescents and Adults.” Social Neuroscience 9, no. 5: 452–470. 10.1080/17470919.2014.933714. 24971880

[brb371864-bib-0138] Sommer, M. , C. Rothmayr , K. Doehnel , et al. 2010. “How Should I Decide? The Neural Correlates of Everyday Moral Reasoning.” Neuropsychologia 48, no. 7: 2018–2026. 10.1016/j.neuropsychologia.2010.03.023. 20362598

[brb371864-bib-0139] Spreng, R. N. , and C. L. Grady . 2010. “Patterns of Brain Activity Supporting Autobiographical Memory, Prospection, and Theory of Mind, and Their Relationship to the Default Mode Network.” Journal of Cognitive Neuroscience 22, no. 6: 1112–1123. 10.1162/jocn.2009.21282. 19580387

[brb371864-bib-0140] Stalnaker, T. A. , N. K. Cooch , M. A. McDannald , T.‐L. Liu , H. Wied , and G. Schoenbaum . 2014. “Orbitofrontal Neurons Infer the Value and Identity of Predicted Outcomes.” Nature Communications 5: Article 3926. 10.1038/ncomms4926. PMC4056018 24894805

[brb371864-bib-0141] Stevens, M. C. , K. A. Kiehl , G. D. Pearlson , and V. D. Calhoun . 2009. “Brain Network Dynamics During Error Commission.” Human Brain Mapping 30, no. 1: 24–37. 10.1002/hbm.20478. PMC2669663 17979124

[brb371864-bib-0142] Strombach, T. , B. Weber , Z. Hangebrauk , et al. 2015. “Social Discounting Involves Modulation of Neural Value Signals by Temporoparietal Junction.” Proceedings of the National Academy of Sciences of the United States of America 112, no. 5: 1619–1624. 10.1073/pnas.1414715112. PMC4321268 25605887

[brb371864-bib-0143] Takahashi, H. , M. Kato , M. Matsuura , et al. 2008. “Neural Correlates of Human Virtue Judgment.” Cerebral Cortex 18, no. 8: 1886–1891. 10.1093/cercor/bhm214. 18203696

[brb371864-bib-0144] Takahashi, Y. K. , M. R. Roesch , R. C. Wilson , et al. 2011. “Expectancy‐Related Changes in Firing of Dopamine Neurons Depend on Orbitofrontal Cortex.” Nature Neuroscience 14, no. 12: 1590‐U1129. 10.1038/nn.2957. PMC3225718 22037501

[brb371864-bib-0145] Tan, H. , Q. Duan , Y. Liu , X. Qiao , and S. Luo . 2022. “Does Losing Money Truly Hurt? The Shared Neural Bases of Monetary Loss and Pain.” Human Brain Mapping 43, no. 10: 3153–3163. 10.1002/hbm.25840 PMC9189080 35315958

[brb371864-bib-0147] Tsoi, L. , J. A. Dungan , A. Chakroff , and L. L. Young . 2018. “Neural Substrates for Moral Judgments of Psychological Versus Physical Harm.” Social Cognitive and Affective Neuroscience 13, no. 5: 460–470. 10.1093/scan/nsy029. PMC6007614 29718384

[brb371864-bib-0148] Turkeltaub, P. E. , G. F. Eden , K. M. Jones , and T. A. Zeffiro . 2002. “Meta‐Analysis of the Functional Neuroanatomy of Single‐Word Reading: Method and Validation.” Neuroimage 16, no. 3: 765–780. 10.1006/nimg.2002.1131. 12169260

[brb371864-bib-0149] Turkeltaub, P. E. , S. B. Eickhoff , A. R. Laird , M. Fox , M. Wiener , and P. Fox . 2012. “Minimizing Within‐Experiment and Within‐Group Effects in Activation Likelihood Estimation Meta‐Analyses.” Human Brain Mapping 33, no. 1: 1–13. 10.1002/hbm.21186. PMC4791073 21305667

[brb371864-bib-0150] Turner, J. A. , and A. R. Laird . 2012. “The Cognitive Paradigm Ontology: Design and Application.” Neuroinformatics 10, no. 1: 57–66. 10.1007/s12021-011-9126-x. PMC3682219 21643732

[brb371864-bib-0151] van den Heuvel, M. P. , I. Libedinsky , S. Quiroz Monnens , J. Repple , I. Sommer , and L. Cocchi . 2026. “Investigating the Methodological Foundation of Lesion Network Mapping.” Nature Neuroscience 29, no. 5: 1237–1247. 10.1038/s41593-025-02196-7 PMC13156034 41540261

[brb371864-bib-0152] van Gils, S. , T. Otto , and N. Dinartika . 2020. “Better together? The Neural Response to Moral Dilemmas Is Moderated by the Presence of a Close Other.” Journal of Neuroscience, Psychology, and Economics 13, no. 3: 150–163. 10.1037/npe0000126.

[brb371864-bib-0153] Varma, M. M. , S. Zhen , and R. Yu . 2023. “Not all Discounts Are Created Equal: Regional Activity and Brain Networks in Temporal and Effort Discounting.” Neuroimage 280: 120363. 10.1016/j.neuroimage.2023.120363 37673412

[brb371864-bib-0154] Verdejo‐Garcia, A. , O. Contreras‐Rodriguez , F. Fonseca , et al. 2014. “Functional Alteration in Frontolimbic Systems Relevant to Moral Judgment in Cocaine‐Dependent Subjects.” Addiction Biology 19, no. 2: 272–281. 10.1111/j.1369-1600.2012.00472.x. 22784032

[brb371864-bib-0155] Wallis, J. D. , K. C. Anderson , and E. K. Miller . 2001. “Single Neurons in Prefrontal Cortex Encode Abstract Rules.” Nature 411, no. 6840: 953–956. 10.1038/35082081. 11418860

[brb371864-bib-0156] Walton, M. E. , J. T. Devlin , and M. F. S. Rushworth . 2004. “Interactions Between Decision Making and Performance Monitoring Within Prefrontal Cortex.” Nature Neuroscience 7, no. 11: 1259–1265. 10.1038/nn1339. 15494729

[brb371864-bib-0157] Wang, T. , L. Mo , C. Mo , et al. 2015. “Is Moral Beauty Different From Facial Beauty? Evidence From an fMRI Study.” Social Cognitive and Affective Neuroscience 10, no. 6: 814–823. 10.1093/scan/nsu123. PMC4448025 25298010

[brb371864-bib-0158] Wen, X. , J. Xie , J. S. Cant , et al. 2020. “The Neural Correlates of Moral Comparison.” Neuropsychologia 149: 107662. 10.1016/j.neuropsychologia.2020.107662. 33129888

[brb371864-bib-0159] Yarkoni, T. , R. A. Poldrack , T. E. Nichols , D. C. Van Essen , and T. D. Wager . 2011. “Large‐Scale Automated Synthesis of Human Functional Neuroimaging Data.” Nature Methods 8, no. 8: 665–670. 10.1038/nmeth.1635. PMC3146590 21706013

[brb371864-bib-0160] Yoder, K. J. , and J. Decety . 2014. “The Good, the Bad, and the Just: Justice Sensitivity Predicts Neural Response During Moral Evaluation of Actions Performed by Others.” Journal of Neuroscience 34, no. 12: 4161–4166. 10.1523/jneurosci.4648-13.2014. PMC3960462 24647937

[brb371864-bib-0161] Young, L. , A. Bechara , D. Tranel , H. Damasio , M. Hauser , and A. Damasio . 2010. “Damage to Ventromedial Prefrontal Cortex Impairs Judgment of Harmful Intent.” Neuron 65, no. 6: 845–851. 10.1016/j.neuron.2010.03.003. PMC3085837 20346759

[brb371864-bib-0162] Young, L. , F. Cushman , M. Hauser , and R. Saxe . 2007. “The Neural Basis of the Interaction Between Theory of Mind and Moral Judgment.” Proceedings of the National Academy of Sciences of the United States of America 104, no. 20: 8235–8240. 10.1073/pnas.0701408104. PMC1895935 17485679

[brb371864-bib-0163] Young, L. , and R. Saxe . 2009. “An fMRI Investigation of Spontaneous Mental State Inference for Moral Judgment.” Journal of Cognitive Neuroscience 21, no. 7: 1396–1405. 10.1162/jocn.2009.21137. 18823250

[brb371864-bib-0164] Zhen, S. S. , Z. A. Yaple , S. B. Eickhoff , and R. J. Yu . 2022. “To Learn or to Gain: Neural Signatures of Exploration in Human Decision‐Making.” Brain Structure & Function 227, no. 1: 63–76. 10.1007/s00429-021-02389-3. 34596757

