# Supplementary material for: Neural Correlates of Moral Judgment in Dilemma Versus Non‐Dilemma Contexts
Source: Brain Behav. 2026 Oct 21;16(10):e71864. doi: 10.1002/brb3.71864 (PMC13687661; doi:10.1002/brb3.71864)
Supplement: Supplementary file 1 [file BRB3-16-e71864-s013.docx]

Table S15. Results of association and uniformity tests using ‘moral’ as a term in Neurosynth

| **Cluster *#*** | **Brain regions** | **BA** | **MNI coordinate (mm)** | | | **Peak *Z* score** | **Cluster Size**  **(voxels)** |
| --- | --- | --- | --- | --- | --- | --- | --- |
|  |  |  | **x** | **y** | **z** |  |  |
| ***A. Association test*** | | | | | | | |
| 1 | dmPFC (R) | 10 | 4 | 62 | 14 | 7.93 | 455 |
| 2 | TPJ (R) | 39 | 54 | -56 | 22 | 7.18 | 208 |
| 3 | TPJ (L) | 39 | -52 | -62 | 26 | 8.76 | 173 |
| 4 | Precuneus (R) | 7 | 2 | -58 | 34 | 6.17 | 130 |
| 5 | vmPFC (R) | 10 | 6 | 52 | -12 | 6.56 | 107 |
| 6 | OFC (R) | 47 | 40 | 26 | -20 | 7.12 | 46 |
| ***B. Uniformity test*** | | | | | | | |
| 1 | dmPFC (L) | 9 | -2 | 56 | 24 | 11.3 | 1738 |
| 2 | Precuneus (R) | 7 | 2 | -58 | 34 | 10.3 | 885 |
| 3 | Insula (R) | 13 | 34 | 24 | 0 | 10.3 | 670 |
| 4 | TPJ (R) | 39 | 54 | -58 | 22 | 10.3 | 614 |
| 5 | TPJ (L) | 39 | -50 | -62 | 26 | 14.1 | 576 |
| 6 | Insula (L) | 13 | -30 | 26 | -2 | 7.47 | 502 |
| 7 | dlPFC | 6 | 0 | 18 | 52 | 7.47 | 418 |
| 8 | IFG (L) | 9 | -46 | 14 | 26 | 7.47 | 242 |
| 9 | Parahippocampus (L) | 34 | -20 | -2 | -24 | 7.47 | 194 |
| 10 | Amygdala (R) | / | 24 | -2 | -16 | 6.52 | 154 |
| 11 | MTG (L) | 20 | -60 | -38 | -8 | 6.52 | 101 |
| *Notes:* L, Left hemisphere; R, Right hemisphere; BA, Brodmann area; MNI, Montreal Neurological Institute; vmPFC, ventromedial prefrontal cortex; dmPFC, dorsomedial prefrontal cortex; dlPFC, dorsolateral prefrontal cortex; OFC, orbital frontal cortex; TPJ, temporoparietal junction; IFG, inferior frontal gyrus; MTG, middle temporal gyrus. | | | | | | | |
