# Supplementary material for: Neural Correlates of Moral Judgment in Dilemma Versus Non‐Dilemma Contexts
Source: Brain Behav. 2026 Oct 21;16(10):e71864. doi: 10.1002/brb3.71864 (PMC13687661; doi:10.1002/brb3.71864)
Supplement: Supplementary file 2 [file BRB3-16-e71864-s017.docx]

**Table S14.** BrainMap behavioral profiling results on non-dilemma tasks, dilemma moral tasks, and their conjunction.

| **A. Conjunction** | | | | | | | | |
| --- | --- | --- | --- | --- | --- | --- | --- | --- |
| **Domain.sub-domain** | **dmPFC_L** | **OFC_L1** | **OFC_L2** | **PC_L** | **TPJ_R** | **TPJ_L** | **TP_R** | **vmPFC_R** |
| Action.Execution | -0.97 | -1.38 | -1.86 | 0.57 | -1.86 | -1.38 | -2.43 | -1.86 |
| Cognition.Attention | 0.80 | 0.41 | -2.32 | 0.98 | -1.96 | -0.25 | -1.31 | -2.32 |
| Cognition.Language.Semantics | 2.25 | 0.02 | 0.60 | 0.60 | -1.12 | 1.90 | -2.22 | -2.22 |
| Cognition.Language.Speech | 2.13 | 0.58 | -2.04 | -0.06 | -0.87 | 1.55 | -1.39 | -1.39 |
| Cognition.Memory.Expliicit | 1.40 | 0.01 | -0.36 | 2.37 | -0.36 | 0.92 | -1.99 | -0.80 |
| Cognition.Memory.Working. | 1.32 | -0.88 | -1.40 | -1.40 | -2.05 | -1.40 | -1.40 | -2.05 |
| Cognition.Reasoning | 1.89 | -0.71 | -1.63 | 1.07 | -1.63 | 2.24 | -2.24 | -0.71 |
| Cognition.Social Cognition | 2.77 | 1.04 | -1.48 | 2.60 | 1.58 | **4.16^**^** | -0.10 | -0.10 |
| Emotion.Positive.Reward/Gain. | 2.23 | 0.05 | -1.96 | -0.33 | -1.96 | 1.43 | -1.96 | -0.77 |
| **B. Dilemma tasks** | | | | | | | | |
| **Domain. sub-domain** | **dmPFC_L** | **TPJ_L** | **TPJ_R** | **TPJ_R** |  |  |  |  |
| Action. Execution | 2.57 | **3.95^**^** | 2.05 | 2.88 |  |  |  |  |
| Action. Imagination | 1.81 | 2.41 | -0.99 | 0.73 |  |  |  |  |
| Cognition. Language | 0.71 | 2.80 | -0.19 | 0.91 |  |  |  |  |
| Cognition. Reasoning | 0.53 | 0.68 | 0.83 | 2.83 |  |  |  |  |
| Cognition. Social cognition | 0.36 | 0.36 | 1.08 | 2.37 |  |  |  |  |
| **C. Non-dilemma moral tasks** | | | | | | | | |
| **Domain. sub-domain** | **vmPFC_R** | **TP_R** | **OFC_L** | **PC_R** |  |  |  |  |
| Action. Execution | -2.37 | **-3.22^**^** | -2.37 | -0.30 |  |  |  |  |
| Cognition. Attention | -2.15 | -2.94 | -2.40 | 1.78 |  |  |  |  |
| Cognition. Reasoning | -2.06 | -2.96 | -0.45 | 0.50 |  |  |  |  |
| Cognition. Working memory | -1.00 | -2.20 | -1.75 | -0.37 |  |  |  |  |
| Cognition. Semantics language | -1.66 | -2.95 | 1.30 | -0.70 |  |  |  |  |
| Emotion. Reward positive | -2.07 | -2.60 | -1.21 | -0.52 |  |  |  |  |

***Notes:*** ^∗∗^*p* < 0.05, Significant *(|z| ≥ 3.0)*; L, Left hemisphere; R, Right hemisphere; vmPFC, ventromedial prefrontal cortex; dmPFC, dorsomedial prefrontal cortex; OFC, orbital frontal cortex; TPJ, temporoparietal junction; PC, precuneus cortex; TP, temporal pole
